# Safety and Immunogenicity of a Locally Produced Inactivated NDV-HXP-S COVID-19 Vaccine (HXP-GPOVac) Compared with BNT162b2: A Phase II Randomized, Controlled, Double-Blind Noninferiority Trial in Thai Adults

**DOI:** 10.3390/vaccines14060481

**Published:** 2026-05-28

**Authors:** Kriengkrai Prasert, Sutthichai Nakphook, Jiraphut Kittiwatanachod, Kanlaya Sornwong, Suriya Naosri, Passakorn Ongarj, Isariya Techatanawat, Piengthong Narakorn, Somchaiya Surichan, Jorge Flores, Laina D. Mercer, Christina S. Polyak, Bruce L. Innis, Rama Raghunandan, Chakrarat Pittayawonganon, Sopon Iamsirithaworn, Supakit Sirilak, Ponthip Wirachwong, Prabda Praphasiri

**Affiliations:** 1 Nakhon Phanom Provincial Hospital, Nakhon Phanom 48000, Thailand; 2 Faculty of Public Health, Chalermphrakiat Sakon Nakhon Campus, Kasetsart University, Sakon Nakhon 47000, Thailand; 3 Government Pharmaceutical Organization (GPO), Bangkok 10400, Thailand; 4 PATH, Seattle, WA 98103, USA; 5 Office of the Permanent Secretary, Ministry of Public Health, Nonthaburi 11000, Thailand; 6 Health Systems Research Institute, Nonthaburi 11000, Thailand

**Keywords:** COVID-19 vaccine, Phase II clinical trial, Newcastle disease virus-vectored vaccine, mRNA vaccine comparator, cell-mediated immunity, Thailand

## Abstract

**Background/Objectives:** HXP-GPOVac is a locally produced, inactivated Newcastle disease virus-based (NDV-HXP-S) COVID-19 vaccine manufactured in Thailand. This phase II trial compared its safety and immunogenicity with the mRNA vaccine BNT162b2 in adults aged 18–75 years. **Methods:** In this randomized, double-blind, active-controlled trial registered with the Thai Clinical Trials Registry (TCTR20220819003), 300 participants were assigned 3:1 to receive HXP-GPOVac or BNT162b2 on Days 1 and 29. Solicited adverse events (AEs) were recorded for 7 days after each dose, AEs were summarized through 28 days after each dose, and serious adverse events (SAEs), medically attended AEs (MAAEs), and adverse events of special interest (AESIs) were collected through Day 197. Humoral immunogenicity was assessed by pseudovirus 50% neutralization titers (NT_50_) and anti-spike IgG concentrations at baseline, Day 29, Day 43, and Day 197. Seroconversion was defined as a ≥4-fold increase from baseline. A predefined subset underwent interferon-γ (IFN-γ) and interleukin-5 (IL-5) ELISpot assays to assess cell-mediated immune responses. The primary immunogenicity analysis assessed non-inferiority of HXP-GPOVac compared with BNT162b2 based on the NT_50_ geometric mean titer ratio, with a prespecified non-inferiority margin of 0.5. **Results:** Solicited AEs were predominantly mild and occurred more frequently after the first dose in both groups; one or more solicited local or systemic AEs were reported by 23.7% (95% CI: 18.3–29.8) of HXP-GPOVac recipients and 44.7% (95% CI: 33.3–56.6) of BNT162b2 recipients after the first dose. AEs through 28 days after vaccination and SAEs were uncommon; MAAEs occurred in 17.0% of HXP-GPOVac recipients and 22.4% of BNT162b2 recipients, and none were considered related to vaccination. In the HXP-GPOVac group, NT_50_ geometric mean titers increased from 5.6 at baseline to 65.5 at Day 29 and 505 at Day 43, declining to 63.6 at Day 197. Anti-spike IgG geometric mean concentrations rose from 7.5 BAU/mL at baseline to 102.7 BAU/mL at Day 29 and 514.6 BAU/mL at Day 43, decreasing to 61.0 BAU/mL at Day 197. BNT162b2 induced higher antibody levels at all time points. The NT_50_ GMT ratio (HXP-GPOVac/BNT162b2) at Day 43 was 0.51 (95% CI: 0.39–0.67); the lower bound did not exceed the prespecified non-inferiority margin of 0.5, and non-inferiority was not established. Seroconversion rates at Day 43 were 97.6% for HXP-GPOVac and 97.1% for BNT162b2 (neutralizing antibody) and 98.6% and 97.1%, respectively (anti-spike IgG). ELISpot analyses demonstrated increased IFN-γ responses after the second dose without evidence of Th2-dominant skewing. **Conclusions:** HXP-GPOVac was well tolerated and induced substantial humoral and cellular immune responses, with high seroconversion rates and balanced T-cell polarization. Although absolute antibody levels were lower than those induced by BNT162b2 and the prespecified non-inferiority criterion was not met, these findings support continued evaluation of the inactivated NDV-HXP-S vaccine platform.

## 1. Introduction

Coronavirus disease 2019 (COVID-19), caused by severe acute respiratory syndrome coronavirus 2 (SARS-CoV-2), has resulted in substantial global morbidity, mortality, and socioeconomic disruption. Although multiple vaccines have been developed and deployed at unprecedented speed, disparities in vaccine access persist, particularly in low- and middle-income countries. These inequities are driven in part by limitations in global manufacturing capacity, cost, and cold-chain requirements associated with advanced platforms, including mRNA and some viral-vector vaccines [1,2,3].

Thailand has prioritized domestic vaccine production as a key component of national health security and pandemic preparedness. Following avian influenza outbreaks first detected in 2004, the Government Pharmaceutical Organization (GPO) established egg-based influenza-vaccine manufacturing capacity through technology transfer supported by the World Health Organization. This initiative enabled local production of seasonal and pandemic influenza vaccines and laid the foundation for broader efforts to strengthen national vaccine self-reliance and preparedness for emerging infectious disease [4,5,6].

Building on this platform, GPO developed HXP-GPOVac, a locally produced, inactivated Newcastle disease virus (NDV)-based vaccine expressing the prefusion-stabilized HexaPro SARS-CoV-2 spike protein. The HexaPro spike incorporates six proline substitutions that improve prefusion stability, antigen expression, and manufacturability compared with earlier two-proline constructs [7,8]. The NDV-HXP-S platform was developed through collaboration among the Icahn School of Medicine at Mount Sinai, PATH, and the University of Texas at Austin and was specifically designed to enable production using conventional egg-based processes already available in influenza-vaccine facilities [7,8,9,10].

Preclinical studies of NDV-HXP-S vaccines demonstrated robust induction of neutralizing antibodies, protection against SARS-CoV-2 challenge in animal models, and no evidence of vaccine-associated enhanced respiratory disease (VAERD) [9,10]. Early-phase clinical trials of inactivated NDV-HXP-S formulations produced in Thailand and Vietnam reported acceptable safety profiles and strong humoral responses [11,12]. A subsequent randomized comparator-controlled phase II trial further supported the safety and immunogenicity of this platform [13]. In Thailand, a phase I trial of an inactivated NDV-HXP-S vaccine demonstrated favorable tolerability and substantial increases in neutralizing and binding antibodies after two doses, supporting selection of a 10-µg antigen dose for further clinical development [11].

Here we report the results of Study 202, a phase II, randomized, double-blind, active-controlled trial evaluating two doses of HXP-GPOVac compared with the mRNA vaccine, BNT162b2 (Pfizer–BioNTech; Comirnaty) in Thai adults aged 18–75 years. The primary objective was to assess the safety and reactogenicity of HXP-GPOVac. Secondary and exploratory objectives included evaluation of humoral immunogenicity, measured by neutralizing antibody titers and anti-spike IgG concentrations, and evaluation of spike-specific cell-mediated immune responses in a predefined substudy.

## 2. Materials and Methods

### 2.1. Study Oversight

This phase II trial was sponsored by the Government Pharmaceutical Organization (GPO) and funded by the Program Management Unit for Competitiveness (PMUC). The trial was conducted at Nakhon Phanom Provincial Hospital, Thailand, in accordance with the principles of the Declaration of Helsinki and the International Council for Harmonization guideline for Good Clinical Practice (ICH-GCP E6(R2)) [14]. The study protocol and informed consent documents were approved by the Ethics Review Committee for Research in Human Subjects, Ministry of Public Health, Thailand. The trial was registered with the Thai Clinical Trials Registry (TCTR20220819003; registered 19 August 2022). All participants provided written informed consent before any study procedures were performed. An independent Data and Safety Monitoring Board (DSMB) and a Protocol Safety Review Team (PSRT) oversaw trial conduct and participant safety throughout the study, in accordance with the protocol.

### 2.2. Study Design and Participants

This was a phase II, randomized, double-blind, active-controlled trial conducted in adults aged 18–75 years. The trial began on 19 August 2022 and was completed on 15 March 2023. Eligible participants were healthy or had stable chronic medical conditions, as determined by the investigators, and were able to comply with all study procedures and follow-up visits. Key exclusion criteria included prior receipt of any COVID-19 vaccine, history of laboratory-confirmed SARS-CoV-2 infection, pregnancy or lactation, current use of systemic immunosuppressive therapy, clinically unstable chronic disease, or any condition that, in the opinion of the investigator, could pose unacceptable risk or interfere with protocol adherence.

Participants were stratified by age group (18–59 years and 60–75 years) and randomized in a 3:1 ratio to receive HXP-GPOVac or the comparator vaccine, BNT162b2. A total of 300 participants were enrolled and randomized, with 224 assigned to HXP-GPOVac and 76 to the BNT162b2 comparator group.

### 2.3. Randomization and Blinding

The randomization schedule was generated by an unblinded statistician at the Center of Excellence for Biomedical and Public Health Informatics (BIOPHICS), Mahidol University, using computer software with block randomization stratified by age group. Block sizes were 8 for the 18–59-year stratum and 12 for the 60–75-year stratum. Allocation concealment was maintained through a centrally held randomization schedule accessed sequentially by unblinded study staff; study vaccines were prepared and administered in identical-appearing syringes to preserve blinding of participants, investigators, and outcome assessors. The trial was conducted in a double-blind manner. Participants, investigators, and study personnel responsible for safety assessments and immunogenicity testing and data analysis remained blind to treatment allocation. Only designated pharmacists or vaccine administrators, who were not involved in participant assessment, were unblinded and were responsible for preparation and administration of study vaccines according to the randomization list. Study vaccines were prepared and administered in identical-appearing syringes to maintain blinding. Unblinding was permitted only when required for appropriate clinical management of a participant.

### 2.4. Vaccines and Administration

HXP-GPOVac is a locally produced, inactivated Newcastle disease virus-based vaccine expressing the prefusion-stabilized HexaPro SARS-CoV-2 spike protein (NDV-HXP-S), manufactured using egg-based processes analogous to those used for seasonal influenza vaccines [7,8,9,10,11]. Each 0.5 mL intramuscular dose contained 10 µg of spike antigen adsorbed to aluminum hydroxide adjuvant, consistent with the dose selected in the preceding phase I trial conducted in Thailand [11]. The comparator was BNT162b2 (Pfizer–BioNTech [Pfizer Inc., New York, NY, USA; BioNTech SE, Mainz, Germany]; Comirnaty), a lipid-nanoparticle-encapsulated, nucleoside-modified mRNA vaccine encoding the full-length SARS-CoV-2 spike protein, administered as a 30-µg intramuscular dose in 0.3 mL [15,16,17]. Both vaccines were administered intramuscularly into the deltoid muscle on Day 1 and Day 29.

### 2.5. Safety Assessments

Participants were observed on-site for at least 30 min after each vaccination for immediate adverse events. Solicited local (pain, erythema, swelling) and systemic (fever, fatigue, headache, myalgia, arthralgia, nausea, vomiting) adverse events (AEs) were recorded by participants on diary cards for 7 days following each vaccination and reviewed by investigators at the follow-up visits, in line with methods commonly used in early-phase COVID-19 vaccine trials [15,16,17]. AEs occurring within 28 days after each vaccination were collected through Day 28 after each dose.

Serious adverse events (SAEs), medically attended adverse events (MAAEs), and adverse events of special interest (AESIs) were collected from Day 1 through Day 197. AEs were graded for severity according to the Division of AIDS (DAIDS) Table for Grading the Severity of Adult and Pediatric Adverse Events, Version 2.1 (2017) [18], and coded using the Medical Dictionary for Regulatory Activities (MedDRA), version 25.1. When a participant experienced multiple occurrences of the same, the maximum severity grade was used for summary analyses. Investigators assessed the relationship of each AE to study vaccination.

Clinical laboratory safety assessments including hematology and serum chemistry parameters were performed at baseline, Day 8, and Day 36 and summarized descriptively. Abnormal values were graded, and clinical relevance and relatedness to vaccination were assessed by investigators.

### 2.6. Immunogenicity Assessments

Blood samples for humoral immunogenicity were collected at baseline (Day 1), Day 29 (28 days after the first dose), Day 43 (14 days after the second dose), and Day 197 (6 months after the second dose). Anti-spike immunoglobulin G (IgG) concentrations were measured by enzyme-linked immunosorbent assay (ELISA) and expressed as geometric mean concentrations (GMCs) in binding antibody units per milliliter (BAU/mL), calibrated to World Health Organization international standards where applicable. Assay procedures followed methods previously described for NDV-HXP-S vaccines in Thailand [11]. Neutralizing antibody responses were measured using a validated pseudovirus neutralization assay and expressed as 50% neutralization titers (NT_50_). Results were summarized as geometric mean titers (GMTs) with 95% confidence intervals [11]. NT_50_ and anti-spike IgG assays were performed at the central laboratory (Bioassay Laboratory, Translational Health Science and Technology Institute [THSTI], Faridabad, India) within the CEPI Centralized Laboratory Network.

Seroconversion was defined as a ≥4-fold increase from baseline in NT_50_ or anti-spike IgG concentration, consistent with definitions used in early-phase COVID-19 vaccine trials across multiple platforms [15,16,17,19,20,21,22]. Reverse cumulative distribution curves were generated to visualize the distribution of antibody responses by vaccine group.

### 2.7. Cell-Mediated Immunity Assessments

A predefined subset of participants was enrolled in an exploratory cell-mediated immunity substudy. Peripheral blood mononuclear cells (PBMCs) were collected at baseline, Day 43, and Day 197, isolated using standardized procedures, and cryopreserved until analysis. PBMCs were stimulated in vitro with overlapping peptide pools spanning the SARS-CoV-2 spike protein. Interferon-γ (IFN-γ) and interleukin-5 (IL-5) enzyme-linked immunospot (ELISpot) assays were performed, and responses were expressed as spot-forming units (SFU) per 10^6^ PBMCs after subtraction of background counts. Assay procedures were harmonized with those used in the preceding phase I NDV-HXP-S trial conducted in Thailand [11]. These assays were performed at the Department of Immunology, Faculty of Medicine Siriraj Hospital, Mahidol University, Bangkok, Thailand. Ratios of IFN-γ to IL-5 responses were calculated as descriptive indices of Th1/Th2-associated cytokine polarization. Cell-mediated immune analyses were exploratory and were not powered for formal between-group hypothesis testing [10,11,13].

### 2.8. Outcomes

The primary outcome was the incidence and severity of solicited local and systemic adverse events within 7 days of each vaccination. Secondary safety outcomes included adverse events occurring within 28 days after vaccination and the occurrence of serious adverse events (SAEs), medically attended adverse events (MAAEs), and adverse events of special interest (AESIs) through Day 197. Secondary immunogenicity outcomes included geometric mean titers (GMTs) of 50% neutralizing antibody titers (NT_50_), geometric mean concentrations (GMCs) of anti-spike immunoglobulin G (IgG), and seroconversion rates at Day 43. Exploratory immunogenicity outcomes included humoral immune responses at Day 29, Day 43, and Day 197 and cell-mediated immune responses, measured by interferon-γ (IFN-γ) and interleukin-5 (IL-5) ELISpot assays and IFN-γ/IL-5 ratios at Day 43 and Day 197.

### 2.9. Sample Size Determination

Sample size was determined based on both safety and immunogenicity objectives. For safety, assuming 200 evaluable participants receiving HXP-GPOVac, the probability of observing at least one serious or severe adverse event was estimated to be 86.8% if the true event rate exceeded 1%, 99.4% if the rate was 2.5%, and 99.9% if the rate was 5% or higher, based on standard binomial probability calculations [23].

For immunogenicity, the sample size for estimation of seroconversion was calculated using the infinite-population proportion formula, $n\;{=Z}_{\left(1-\alpha /2\right)}^{2}\;p(1-p)/d^{2}$, assuming an expected seroconversion rate (*p*) of 97% based on phase I data [11], an allowable margin of error (*d*) of 0.03, and two-sided *α* of 0.01, yielding a requirement of 215 participants in the HXP-GPOVac group. For the neutralizing antibody geometric mean titer ratio non-inferiority objective, non-inferiority was defined as the lower bound of the two-sided 95% confidence interval (CI) exceeding 0.5, as prespecified in the study protocol. The null hypothesis for the non-inferiority test was that the NT_50_ GMT ratio (HXP-GPOVac/BNT162b2) was less than 0.5; the alternative hypothesis was that the ratio was at least 0.5. Non-inferiority would be declared if the lower bound of the two-sided 95% CI for the GMT ratio exceeded 0.5. Assuming a true GMT ratio of 1.0, a coefficient of variation of 0.6, a 3:1 allocation ratio, 90% power, and one-sided *α* of 0.025, the required total sample size was 300 participants (225 assigned to HXP-GPOVac and 75 to BNT162b2). This was operationalized as 224 and 76 participants, respectively.

### 2.10. Statistical Analysis

All statistical analyses were prespecified in the Statistical Analysis Plan and performed using SAS^®^ version 9.4 (SAS Institute, Cary, NC, USA). Analyses were primarily descriptive, with no adjustment for multiplicity. Given the phase II nature of the study and the prespecified exploratory character of secondary and subgroup analyses, formal multiplicity adjustment was not applied, consistent with the descriptive, phase II nature of this study. Demographic and baseline characteristics were summarized by treatment group. Continuous variables were summarized using mean, standard deviation, median, minimum and maximum values, and categorical variables using counts and percentages. Between-group comparisons were conducted using chi-square tests, independent *t* tests, or Wilcoxon rank-sum tests, as appropriate.

Safety analyses were performed in the safety population according to treatment received. The proportions of participants experiencing solicited AEs, AEs occurring within 28 days after vaccination, SAEs, MAAEs, and AESIs were summarized with two-sided 95% confidence intervals calculated using the Clopper–Pearson exact method [23].

Immunogenicity analyses were conducted primarily in the per-protocol population, with analyses in the full analysis set performed as supportive. The Full Analysis Set followed the intention-to-treat principle, comprising all randomized participants analyzed according to their assigned treatment group. NT_50_ titers and anti-spike IgG concentrations were log-transformed and summarized as GMTs and GMCs with 95% confidence intervals at each time point (calculated on the log scale and back-transformed). Geometric mean fold rises (GMFRs) from baseline were calculated with corresponding confidence intervals. Seroconversion rates were summarized with two-sided 95% confidence intervals using the Clopper–Pearson method [23]. Ratios of GMTs and GMCs between vaccine groups were calculated with 95% confidence intervals. Prespecified subgroup analysis included age strata (18–59 years and 60–75 years) and participants without documented symptomatic COVID-19 infection or receipt of additional COVID-19 vaccines during follow-up. At enrollment, prior SARS-CoV-2 infection was excluded through a positive COVID-19 test history and a negative Rapid Antigen Test confirmed by RT-PCR; baseline serological testing was not used as an eligibility criterion. The low baseline NT_50_ GMTs (5.62 and 6.45 in the HXP-GPOVac and BNT162b2 groups, respectively) are consistent with a predominantly seronegative population at enrollment. Participants with seropositive baseline titers were excluded from the per-protocol GMT analysis per prespecified criteria. All immunogenicity analyses were performed on observed data without formal imputation. Participants with missing data at a given time point were excluded from analyses at that time point. The proportion of participants with missing immunogenicity data was small, and no systematic differential patterns of missingness were identified between treatment groups. Analyses assumed data were missing at random (MAR).

## 3. Results

### 3.1. Study Enrollment and Baseline Characteristics

**Figure 1:**
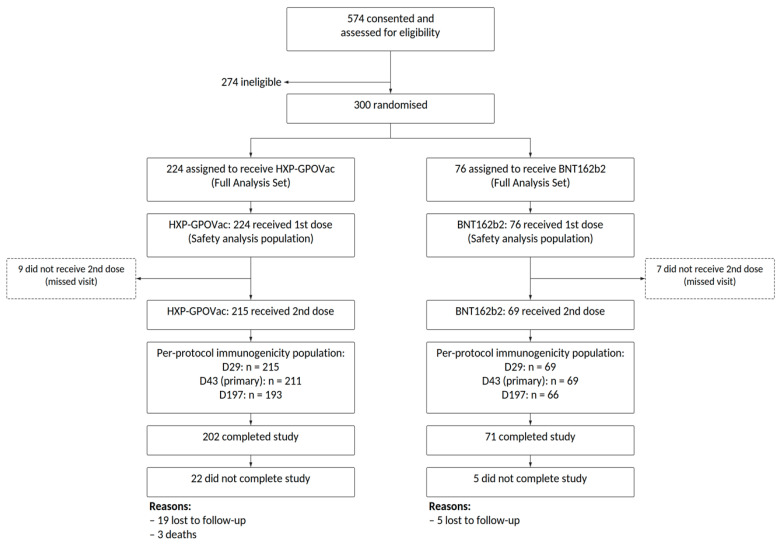
Disposition of study participants throughout the study. Dashed boxes indicate participants who did not receive the second dose owing to a missed visit.

Enrollment began on 19 August 2022 and was completed on 25 August 2022. A total of 574 individuals were screened for eligibility, of whom 300 were enrolled and randomized to receive two doses of HXP-GPOVac (*n* = 224) or BNT162b2 (*n* = 76) (Figure 1). All randomized participants received at least one dose of study vaccine and were included in the safety population. Baseline demographic and clinical characteristics were similar between the two groups (Table 1). Overall, 77.7% of participants were aged 18–59 years and 22.3% were aged 60–75 years. The mean age was 48.1 years (standard deviation, 13.9), and 75.7% of participants were male. All participants self-reported Asian race. Mean body mass index and the distribution of underlying medical conditions were similar between groups, with no clinically meaningful differences observed. These findings indicate that the randomized groups were comparable at enrollment, supporting the validity of subsequent safety and immunogenicity comparisons.

### 3.2. Safety and Reactogenicity

Solicited local adverse events during the 7 days following the first vaccination were reported by 23.7% of participants who received HXP-GPOVac and 44.7% of those who received BNT162b2 (Table 2). After the second vaccination, solicited local reactions were reported by 17.8% and 24.6% of participants in the HXP-GPOVac and BNT162b2 groups, respectively. Injection-site pain or tenderness was the most frequently reported local reaction in both groups. Swelling and erythema were infrequent, and no Grade 4 local reactions were observed. Solicited systemic adverse events after the first dose occurred in 29.9% of HXP-GPOVac recipients and 36.8% of BNT162b2 recipients, and after the second dose in 20.1% and 23.2%, respectively. The most frequently reported systemic events were fatigue or malaise, myalgia, headache, and arthralgia. Across both vaccine groups and study time points, the majority of solicited reactions were Grade 1 or Grade 2 in severity, with occasional Grade 3 events and no Grade 4 events reported. For both vaccines, solicited reactions were reported more frequently after the first dose than after the second dose. Patterns of reactogenicity were generally consistent across age strata (Table 2 and Table S1). Solicited adverse event severity grading criteria are provided in Table S2.

AEs occurring within 28 days after vaccination were reported in 10.3% of HXP-GPOVac recipients and 11.8% of BNT162b2 recipients after the first dose, and in 4.7% and 7.2%, respectively, after the second dose (Table 3). Medically attended adverse events occurred in 17.0% of HXP-GPOVac recipients and 22.4% of BNT162b2 recipients during the study period (Table 3). Seven participants experienced serious adverse events: five (2.2%) in the HXP-GPOVac recipients and two (2.6%) in the BNT162b2 group. Three deaths occurred among HXP-GPOVac recipients during follow-up, all occurring more than 100 days after the second dose. Two participants (aged 49 and 55 years) experienced unattended deaths at Day 104 and Day 163 post-second dose, respectively; no preceding clinical events were recorded. One participant (aged 75 years) experienced subarachnoid hemorrhage, infective endocarditis, and bacterial pneumonia beginning approximately 126 days after the second dose, resulting in death on Day 128; unstable supraventricular tachycardia also occurred and resolved. The temporal separation from vaccination, absence of biological plausibility, and the nature of the events were the primary bases for causality assessments. All deaths were assessed by investigators and the independent Data and Safety Monitoring Board as unrelated to study vaccination. No adverse events of special interest were identified in either vaccine group. Clinical laboratory assessments of hematology and serum chemistry performed at baseline, Day 8, and Day 36 revealed no clinically significant changes from baseline in any parameter, including hemoglobin, white blood cell count, platelets, creatinine, ALT, AST, or total bilirubin. Abnormal values were observed in approximately 30% of participants for hematology and 20% for chemistry; almost all were Grade 1, with a small number of Grade 2 findings. No laboratory abnormalities were attributed to vaccination. Detailed results are provided in Supplementary Tables S5 and S6. Overall, both vaccines were generally well tolerated, with predominantly mild to moderate reactogenicity, low rates of serious adverse events, and no vaccine-related medically attended adverse events.

### 3.3. Immunogenicity

**Figure 2:**
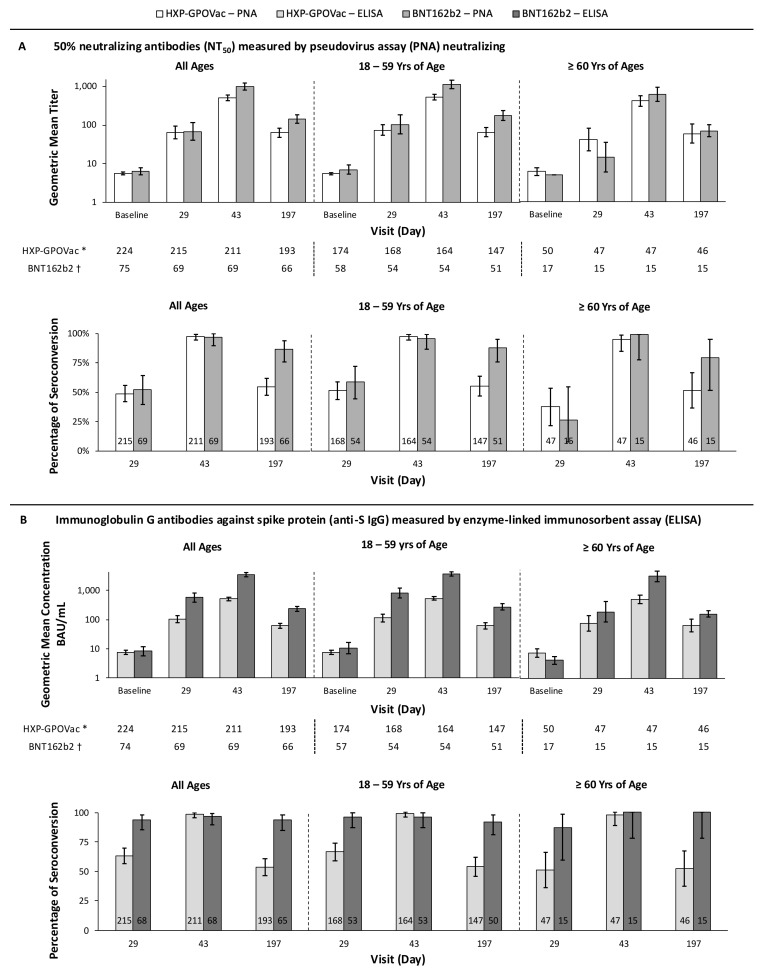
Geometric mean titers or concentrations and seroconversion rates for (**A**) 50% neutralizing antibody titers and (**B**) anti-spike immunoglobulin G concentrations at baseline, 28 days after the first dose (D29), 14 days after the second dose (D43), and 6 months after the second dose (D197), by randomization arm and age stratum (per-protocol population). Abbreviations: anti-spike IgG, anti-spike immunoglobulin G; BAU/mL, binding antibody units per milliliter; ELISA, enzyme-linked immunosorbent assay; GMC, geometric mean concentration; GMT, geometric mean titer; NT_50_, 50% neutralization titer; PNA, pseudovirus neutralization assay. Notes: Numbers of participants with no missing data in both the HXP-GPOVac and BNT162b2 groups at different time points are shown in the tables below the graphs. Error bars represent 95% CIs. * indicates n for the HXP-GPOVac group at each visit. † indicates n for the BNT162b2 group at each visit.

Baseline neutralizing antibody titers and anti-spike IgG concentrations were low in both groups (Figure 2 and Table S3). In the per-protocol population, baseline NT_50_ geometric mean titers (GMTs) were 5.62 in the HXP-GPOVac group and 6.45 in the BNT162b2 group, and baseline anti-spike IgG geometric mean concentrations (GMCs) were 7.45 and 8.35 BAU/mL, respectively. By Day 29 (28 days after the first dose), neutralizing antibody responses increased in both groups. NT_50_ GMTs rose to 65.52 in the HXP-GPOVac group and 67.80 in the BNT162b2 group, corresponding to seroconversion rates of 48.8% and 52.2%, respectively. Anti-spike IgG GMCs at Day 29 were 102.73 BAU/mL in the HXP-GPOVac group and 579.53 BAU/mL in the BNT162b2 group, with IgG seroconversion rates of 63.3% and 94.1%, respectively.

Fourteen days after the second dose (Day 43), both vaccines induced marked increases in humoral immune responses (Figure 2 and Table S3). NT_50_ GMTs increased to 504.97 in the HXP-GPOVac group and 993.34 in the BNT162b2 group. Seroconversion rates for neutralizing antibodies exceeded 97% in both groups. Anti-spike IgG GMCs at Day 43 were 514.58 BAU/mL for HXP-GPOVac and 3448.32 BAU/mL for BNT162b2, with IgG seroconversion rates of 98.6% and 97.1%, respectively. At Day 197 (6 months after the second dose), neutralizing antibody titers and IgG concentrations had declined relative to peak post-second dose levels but remained above baseline values in both groups. NT_50_ GMTs were 63.56 in the HXP-GPOVac group and 142.36 in the BNT162b2 group, with corresponding seroconversion rates of 54.9% and 86.4%. Anti-spike IgG GMCs at Day 197 were 61.01 BAU/mL and 237.57 BAU/mL, respectively, and IgG seroconversion rates were 53.4% and 93.8%. Geometric mean ratios for NT_50_ GMTs and anti-spike IgG GMCs across time points are presented in Table 4. The NT_50_ GMT ratio (HXP-GPOVac/BNT162b2) at Day 43 was 0.51 (95% CI: 0.39–0.67); the lower bound of 0.39 did not exceed the prespecified non-inferiority margin of 0.5, and non-inferiority was not established.

Age-stratified analyses showed similar patterns of response in adults aged 18–59 years and 60–75 years, with lower absolute antibody levels observed among older participants in both vaccine groups (Table S3). Reverse cumulative distribution curves demonstrated rightward shifts in antibody responses following each dose for both vaccines, with higher distributions observed for BNT162b2 at all post-vaccination time points (Figure 2).

### 3.4. Cell-Mediated Immunity

**Figure 3:**
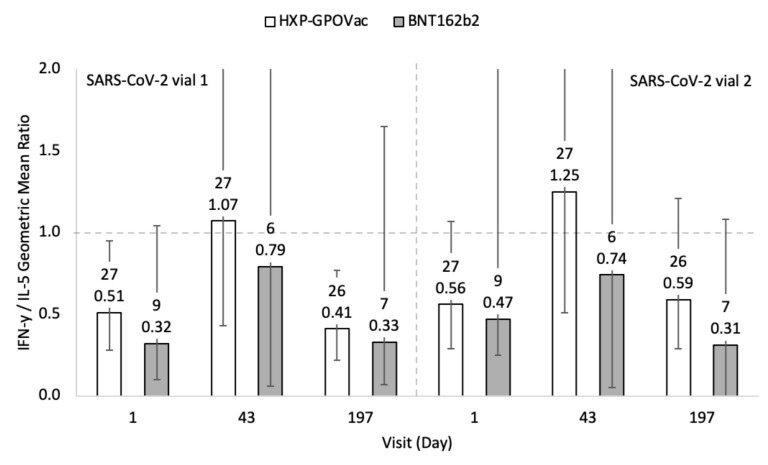
Cell-mediated immune responses to SARS-CoV-2 spike: IFN-γ/IL-5 ELISpot geometric mean ratios in HXP-GPOVac and BNT162b2 recipients at baseline (D1), 14 days after the second dose (D43), and 6 months after the second dose (D197), using SARS-CoV-2 peptide vial 1 and vial 2. Abbreviations: IFN-γ, interferon gamma; IL-5, interleukin-5; ELISpot, enzyme-linked immunospot assay; GMR, geometric mean ratio; CI, confidence interval. Notes: IFN-γ/IL-5 ratio reflects the balance of Th1-biased (IFN-γ-dominant) versus Th2-associated (IL-5-dominant) cytokine responses. Vial 1 and Vial 2 correspond to two separate overlapping peptide pools spanning the SARS-CoV-2 spike protein. Sample sizes differ between timepoints due to the predefined CMI subset and attrition across visits. Values <1 suggest relatively higher IL-5 activity, whereas values >1 indicate a Th1-leaning cytokine profile. Error bars represent 95% CIs.

Cell-mediated immune responses were evaluated in a predefined exploratory subset of participants (Table S4). At baseline, interferon-γ (IFN-γ) ELISpot responses were low in both vaccine groups. Following vaccination, IFN-γ responses increased at Day 43 in recipients of both HXP-GPOVac and BNT162b2 and declined by Day 197 toward baseline levels (Figure 3). Interleukin-5 (IL-5) ELISpot responses remained relatively stable over time in both groups, with modest changes observed after vaccination. As a result, the ratio of IFN-γ to IL-5 increased from baseline to Day 43 in both vaccine groups and decreased by Day 197, indicating transient enhancement of Th1-associated cytokine responses following the second dose (Figure 3). Across time points, IFN-γ/IL-5 ratios were of similar magnitude between vaccine groups, and no sustained Th2-dominant cytokine pattern was observed. Peripheral blood mononuclear cell yields were consistent across visits and between groups. Given the exploratory nature of the substudy and the limited sample size, these findings are descriptive.

## 4. Discussion

In this phase II, randomized, double-blind, active-controlled trial conducted in Thai adults, the locally produced inactivated NDV-HXP-S COVID-19 vaccine HXP-GPOVac demonstrated an acceptable safety profile and induced substantial humoral and cellular immune responses following a two-dose primary series. Although absolute neutralizing antibody levels were lower than those induced by BNT162b2 and the prespecified non-inferiority criterion was not met, high seroconversion rates were achieved in both groups. Reactogenicity was predominantly mild to moderate, serious adverse events were uncommon, and no events were considered related to vaccination.

Consistent with prior reports of inactivated NDV-HXP-S vaccines, HXP-GPOVac elicited substantial increases in neutralizing antibody titers and binding antibody concentrations after priming and boosting [9,10,11,12,13]. Although geometric mean antibody levels were lower than those induced by the mRNA comparator BNT162b2, seroconversion rates exceeded 95% in both vaccine groups at Day 43. This pattern, high responder proportions with comparatively lower peak antibody magnitudes, is characteristic of inactivated SARS-CoV-2 vaccines and has been observed with other licensed products, including CoronaVac [19,21]. Importantly, a ≥4-fold rise from baseline reflects a meaningful immunologic response in early-phase trials and is widely used across vaccine platforms as a pragmatic measure of immunogenicity rather than a correlate of clinical protection [17,19,21].

Antibody levels declined by six months after the second dose in both groups, consistent with the waning observed following primary COVID-19 vaccination with inactivated and mRNA platforms [24]. The pattern of decline from Day 43 to Day 197 was observed in both vaccine groups, with BNT162b2 maintaining higher absolute levels throughout follow-up. Despite this decline, antibody concentrations remained above baseline values at Day 197. The clinical implications of waning antibody levels remain complex and are influenced by additional factors, including immune memory, variant-specific antigenic differences, and subsequent boosting. Correlates-of-risk analyses indicate that neutralizing and binding antibodies are quantitative, but not absolute, predictors of protection against symptomatic infection [25,26]. This study was not designed to assess durability of protection or booster responses, and longer-term follow-up and additional studies are warranted.

Exploratory analyses of cell-mediated immunity demonstrated increased IFN-γ ELISpot responses following the second dose, with relatively stable IL-5 responses, resulting in transient increases in IFN-γ/IL-5 ratios. These findings indicate induction of spike-specific T-cell activity without evidence of sustained Th2-dominant skewing. Th1-associated responses characterized by IFN-γ production are generally considered supportive of antiviral immunity, whereas disproportionate Th2 responses have been implicated in vaccine-associated enhanced respiratory disease in historical respiratory virus vaccine models [27]. In this trial, no adverse events of special interest or clinical features suggestive of enhanced disease were observed. Given the exploratory nature of the cell-mediated immunity substudy, the limited sample size, and the absence of deeper T-cell phenotyping such as CD4/CD8 characterization or functional profiling, these findings should be interpreted descriptively and do not support formal conclusions regarding the magnitude or quality of T-cell responses.

The favorable safety and immunogenicity profile observed in this study builds on prior preclinical and early-phase clinical evaluations of the NDV-HXP-S platform conducted in multiple settings [9,10,11,12,13]. A key feature of this vaccine approach is its compatibility with established egg-based manufacturing infrastructure, which may facilitate local production and improve access in settings where advanced vaccine platforms remain costly or logistically challenging [4,5,7,8,9,10]. Domestic production capacity can contribute to national pandemic preparedness and resilience by reducing reliance on global supply chains during public health emergencies [6].

This study has several limitations. As a phase II trial, it was not powered to evaluate clinical efficacy, and infection outcomes were not prespecified endpoints. Immunogenicity assays were based on the ancestral SARS-CoV-2 strain, and variant-specific neutralization against currently circulating strains was not assessed, limiting direct extrapolation to protection against variants with substantial antigenic divergence [25,26]. The cell-mediated immunity analyses were conducted in a predefined subset of participants, were exploratory in nature, and lacked deeper T-cell characterization such as CD4/CD8 phenotyping or functional profiling. The study enrolled a predominantly male population (75.7%), which may limit generalizability across sexes. All participants self-reported Asian race, and the trial was conducted at a single site in Thailand, restricting ethnic and geographic diversity. The relatively small sample size, particularly in the BNT162b2 comparator group (*n* = 76), limits the precision of between-group comparisons. Loss to follow-up at Day 197 was 8.5% in the HXP-GPOVac group and 6.6% in the BNT162b2 group; age-stratified loss to follow-up data were not systematically analyzed, and differential attrition by age stratum or comorbidity cannot be fully excluded as a potential source of bias in the Day 197 immunogenicity estimates. Participants with significant immunocompromise were excluded, and findings may not apply to immunocompromised populations. In summary, two doses of HXP-GPOVac were well tolerated and induced substantial humoral and cellular immune responses, including high seroconversion rates and measurable spike-specific T-cell activity without evidence of Th2-dominant polarization. However, absolute neutralizing antibody levels were significantly lower than those induced by BNT162b2, and the prespecified non-inferiority criterion for the NT_50_ GMT ratio was not met. These results should be interpreted in the context of a phase II trial not powered for efficacy. The favorable safety profile and immunogenic activity of HXP-GPOVac support further evaluation of the inactivated NDV-HXP-S platform, including assessment of higher antigen doses, adjuvant optimization, booster strategies, and variant-adapted formulations before advancement to later-phase studies.

## Figures and Tables

**Table 1:** Baseline characteristics of study participants by randomization arm (safety population).

	All (*N* = 300)	Vaccine Group	
		HXP-GPOVac (*N* = 224)	BNT162b2 (*N* = 76)
Age—yrs			
*n*	300	224	76
Mean (SD)	48.1 (13.9)	49.0 (13.3)	45.7 (15.4)
Median (q1–q3)	48.0 (39.0–57.5)	49.0 (40.5–57.5)	47.0 (35.5–57.5)
Min, Max	18, 75	19, 75	18, 75
Sex—*n* (%)			
Male	227 (75.7)	172 (76.8)	55 (72.4)
Female	73 (24.3)	52 (23.2)	21 (27.6)
Race—*n* (%)			
Asian	300 (100)	224 (100)	76 (100)
Height—cm			
*N*	300	224	76
Mean (SD)	159.9 (7.7)	160.2 (7.8)	159.2 (7.5)
Median (q1–q3)	160.0 (155.0–165.0)	160.0 (155.0–165.0)	161.0 (154.5–165.0)
Min, Max	137, 181	137, 181	140, 175
Weight—kg			
*N*	300	224	76
Mean (SD)	58.8 (9.6)	58.7 (9.8)	58.9 (9.2)
Median (q1–q3)	57.6 (51.8–64.5)	57.5 (51.4–64.5)	57.9 (52.0–64.7)
Min, Max	41.6, 89	41.6, 89	44.1, 85
Body Mass Index (BMI)—kg/m^2^			
*n*	300	224	76
Mean (SD)	23.0 (3.4)	22.9 (3.3)	23.3 (3.6)
Median (q1–q3)	22.4 (20.5–25.0)	22.2 (20.4–24.9)	22.6 (20.7–25.8)
Min, Max	17.1, 30.0	17.1, 29.9	17.3, 30.0
Medical History by System Organ Class (SOC) and Preferred Term (PT)—NE (%)			
NE	30	16	14
All SOC			
All PT—NE (%)	30 (100)	16 (100)	14 (100)
Cardiac disorders			
Myocardial ischaemia	1 (3.3)	0 (0)	1 (7.1)
Endocrine disorders			
Diabetes Mellitus	5 (16.7)	3 (18.8)	2 (14.3)
Eye disorders			
Cataract	1 (3.3)	1 (6.3)	0 (0)
Conjunctivitis	1 (3.3)	1 (6.3)	0 (0)
Immune system disorders			
Asthma	2 (6.7)	1 (6.3)	1 (7.1)
Injury, poisoning and procedural complications			
Post-traumatic epilepsy	1 (3.3)	1 (6.3)	0 (0)
Metabolism and nutrition disorders			
Dyslipidaemia	1 (3.3)	1 (6.3)	0 (0)
Reproductive system and breast disorders			
Benign prostatic hyperplasia	1 (3.3)	1 (6.3)	0 (0)
Respiratory, thoracic and mediastinal disorders			
Chronic obstructive pulmonary disease	1 (3.3)	1 (6.3)	0 (0)
Surgical and medical procedures			
Percutaneous coronary intervention	1 (3.3)	0 (0)	1 (7.1)
Vascular disorders			
Cerebral infarction	1 (3.3)	0 (0)	1 (7.1)
Hypertension	14 (46.7)	6 (37.5)	8 (57.1)

Abbreviations: SD, standard deviation; BMI, body mass index; Q1–Q3, interquartile range; SOC, System Organ Class; PT, Preferred Term. Note: Data are presented as *n* (%), mean (SD), or median (Q1–Q3). Percentages are based on the number of participants with available data. “NE” represents the number of medical history events.

**Table 2:** Incidence of solicited local and systemic adverse events during the first 7 days after each vaccination, by randomization arm and age stratum (safety population).

	Overall		18–59 yrs		≥60 yrs	
	HXP-GPOVac (*N* = 224)	BNT162b2 (*N* = 76)	HXP-GPOVac (*N* = 174)	BNT162b2 (*N* = 59)	HXP-GPOVac (*N* = 50)	BNT162b2 (*N* = 17)
Solicited local adverse events						
1st Dose—*n*	224	76	174	59	50	17
Any local reaction—*n* (%)	53 (23.7)	34 (44.7)	46 (26.4)	28 (47.5)	7 (14.0)	6 (35.3)
(95% CI)	(18.3–29.8)	(33.3–56.6)	(20.1–33.6)	(34.3–60.9)	(5.8–26.7)	(14.2–61.7)
2nd Dose—*n*	214	69	167	54	47	15
Any local reaction—*n* (%)	38 (17.8)	17 (24.6)	32 (19.2)	13 (24.1)	6 (12.8)	4 (26.7)
(95% CI)	(12.9–23.5)	(15.1–36.5)	(13.5–26.0)	(13.5–37.6)	(4.8–25.7)	(7.8–55.1)
Pain or Tenderness						
1st Dose—*n*	224	76	174	59	50	17
With one or more—*n* (%)	51 (22.8)	34 (44.7)	44 (25.3)	28 (47.5)	7 (14.0)	6 (35.3)
(95% CI)	(17.4–28.8)	(33.3–56.6)	(19.0–32.4)	(34.3–60.9)	(5.8–26.7)	(14.2–61.7)
2nd Dose—*n*	214	69	167	54	47	15
With one or more—*n* (%)	38 (17.8)	17 (24.6)	32 (19.2)	13 (24.1)	6 (12.8)	4 (26.7)
(95% CI)	(12.9–23.5)	(15.1–36.5)	(13.5–26.0)	(13.5–37.6)	(4.8–25.7)	(7.8–55.1)
Swelling or Induration						
1st Dose—*n*	224	76	174	59	50	17
With one or more—*n* (%)	2 (0.9)	0 (0)	2 (1.1)	0 (0)	0 (0)	0 (0)
(95% CI)	(0.1–3.2)	(0.0–4.7)	(0.1–4.1)	(0.0–6.1)	(0.0–7.1)	(0.0–19.5)
2nd Dose—*n*	214	69	167	54	47	15
With one or more—*n* (%)	0 (0)	0 (0)	0 (0)	0 (0)	0 (0)	0 (0)
(95% CI)	(0.0–1.7)	(0.0–5.2)	(0.0–2.2)	(0.0–6.6)	(0.0–7.5)	(0.0–21.8)
Erythema						
1st Dose—*n*	224	76	174	59	50	17
With one or more—*n* (%)	0 (0)	2 (2.6)	0 (0)	2 (3.4)	0 (0)	0 (0)
(95% CI)	(0.0–1.6)	(0.3–9.2)	(0.0–2.1)	(0.4–11.7)	(0.0–7.1)	(0.0–19.5)
2nd Dose—*n*	214	69	167	54	47	15
With one or more—*n* (%)	0 (0)	0 (0)	0 (0)	0 (0)	0 (0)	0 (0)
(95% CI)	(0.0–1.7)	(0.0–5.2)	(0.0–2.2)	(0.0–6.6)	(0.0–7.5)	(0.0–21.8)
Solicited systemic adverse events						
1st Dose—*n*	224	76	174	59	50	17
Any systemic reaction—*n* (%)	67 (29.9)	28 (36.8)	62 (35.6)	25 (42.4)	5 (10.0)	3 (17.6)
(95% CI)	(24.0–36.4)	(26.1–48.7)	(28.5–43.2)	(29.6–55.9)	(3.3–21.8)	(3.8–43.4)
2nd Dose—*n*	214	69	167	54	47	15
Any systemic reaction—*n* (%)	43 (20.1)	16 (23.2)	37 (22.2)	13 (24.1)	6 (12.8)	3 (20.0)
(95% CI)	(14.9–26.1)	(13.9–34.9)	(16.1–29.2)	(13.5–37.6)	(4.8–25.7)	(4.3–48.1)
Fever (≥38 °C)						
1st Dose—*n*	224	76	174	59	50	17
With one or more—*n* (%)	0 (0)	0 (0)	0 (0)	0 (0)	0 (0)	0 (0)
(95% CI)	(0.0–1.6)	(0.0–4.7)	(0.0–2.1)	(0.0–6.1)	(0.0–7.1)	(0.0–19.5)
2nd Dose—*n*	214	69	167	54	47	15
With one or more—*n* (%)	0 (0)	0 (0)	0 (0)	0 (0)	0 (0)	0 (0)
(95% CI)	(0.0–1.7)	(0.0–5.2)	(0.0–2.2)	(0.0–6.6)	(0.0–7.5)	(0.0–21.8)
Headache						
1st Dose—*n*	224	76	174	59	50	17
With one or more—*n* (%)	33 (14.7)	16 (21.1)	31 (17.8)	14 (23.7)	2 (4.0)	2 (11.8)
(95% CI)	(10.4–20.1)	(12.5–31.9)	(12.4–24.3)	(13.6–36.6)	(0.5–13.7)	(1.5–36.4)
2nd Dose—*n*	214	69	167	54	47	15
With one or more—*n* (%)	23 (10.7)	7 (10.1)	20 (12.0)	5 (9.3)	3 (6.4)	2 (13.3)
(95% CI)	(6.9–15.7)	(4.2–19.8)	(7.5–17.9)	(3.1–20.3)	(1.3–17.5)	(1.7–40.5)
Fatigue or Malaise						
1st Dose—*n*	224	76	174	59	50	17
With one or more—*n* (%)	38 (17.0)	14 (18.4)	35 (20.1)	14 (23.7)	3 (6.0)	0 (0)
(95% CI)	(12.3–22.5)	(10.5–29.0)	(14.4–26.8)	(13.6–36.6)	(1.3–16.5)	(0.0–19.5)
2nd Dose—*n*	214	69	167	54	47	15
With one or more—*n* (%)	29 (13.6)	9 (13.0)	25 (15.0)	7 (13.0)	4 (8.5)	2 (13.3)
(95% CI)	(9.3–18.9)	(6.1–23.3)	(9.9–21.3)	(5.4–24.9)	(2.4–20.4)	(1.7–40.5)
Myalgia						
1st Dose—*n*	224	76	174	59	50	17
With one or more—*n* (%)	47 (21.0)	19 (25.0)	43 (24.7)	18 (30.5)	4 (8.0)	1 (5.9)
(95% CI)	(15.8–26.9)	(15.8–36.3)	(18.5–31.8)	(19.2–43.9)	(2.2–19.2)	(0.1–28.7)
2nd Dose—*n*	214	69	167	54	47	15
With one or more—*n* (%)	31 (14.5)	13 (18.8)	28 (16.8)	12 (22.2)	3 (6.4)	1 (6.7)
(95% CI)	(10.1–19.9)	(10.4–30.1)	(11.4–23.3)	(12.0–35.6)	(1.3–17.5)	(0.2–31.9)
Arthralgia						
1st Dose—*n*	224	76	174	59	50	17
With one or more—*n* (%)	26 (11.6)	5 (6.6)	24 (13.8)	5 (8.5)	2 (4.0)	0 (0)
(95% CI)	(7.7–16.5)	(2.2–14.7)	(9.0–19.8)	(2.8–18.7)	(0.5–13.7)	(0.0–19.5)
2nd Dose—*n*	214	69	167	54	47	15
With one or more—*n* (%)	22 (10.3)	3 (4.3)	20 (12.0)	2 (3.7)	2 (4.3)	1 (6.7)
(95% CI)	(6.6–15.2)	(0.9–12.2)	(7.5–17.9)	(0.5–12.7)	(0.5–14.5)	(0.2–31.9)
Nausea or Vomiting						
1st Dose—*n*	224	76	174	59	50	17
With one or more—*n* (%)	7 (3.1)	3 (3.9)	7 (4.0)	3 (5.1)	0 (0)	0 (0)
(95% CI)	(1.3–6.3)	(0.8–11.1)	(1.6–8.1)	(1.1–14.1)	(0.0–7.1)	(0.0–19.5)
2nd Dose—*n*	214	69	167	54	47	15
With one or more—*n* (%)	5 (2.3)	1 (1.4)	5 (3.0)	1 (1.9)	0 (0)	0 (0)
(95% CI)	(0.8–5.4)	(0.0–7.8)	(1.0–6.8)	(0.0–9.9)	(0.0–7.5)	(0.0–21.8)

Abbreviations: AE, adverse event; CI, confidence interval; °C, degrees Celsius. Note: Data are *n* (%) of participants with ≥1 event. Denominators are the number vaccinated at that dose; 95% CIs are exact (Clopper–Pearson). Dose labels are standardized as “1st Dose” and “2nd Dose”.

**Table 3:** Incidence of adverse events and severity during the first 28 days after each vaccination, and serious adverse events, medically attended adverse events, and adverse events of special interest during the entire study period (Day 1–197), by randomization arm and age stratum (safety population).

	Overall		18–59 yrs		≥60 yrs	
	HXP-GPOVac	BNT162b2	HXP-GPOVac	BNT162b2	HXP-GPOVac	BNT162b2
	(*N* = 224)	(*N* = 76)	(*N* = 174)	(*N* = 59)	(*N* = 50)	(*N* = 17)
Overall adverse events (AEs)						
1st Dose—*n*	224	76	174	59	50	17
With one or more AEs—*n* (%)	23 (10.3)	9 (11.8)	17 (9.8)	7 (11.9)	6 (12.0)	2 (11.8)
(95% CI)	(6.6–15.0)	(5.6–21.3)	(5.8–15.2)	(4.9–22.9)	(4.5–24.3)	(1.5–36.4)
Mild (Grade 1)—*n* (%)	21 (9.4)	6 (7.9)	16 (9.2)	5 (8.5)	5 (10.0)	1 (5.9)
Moderate (Grade 2)—*n* (%)	2 (0.9)	1 (1.3)	1 (0.6)	1 (1.7)	1 (2.0)	0 (0)
Severe (Grade 3)—*n* (%)	0 (0)	2 (2.6)	0 (0)	1 (1.7)	0 (0)	1 (5.9)
Potentially life-threatening (Grade 4)—*n* (%)	0 (0)	0 (0)	0 (0)	0 (0)	0 (0)	0 (0)
With vaccine-related AEs—*n* (%)	0 (0)	0 (0)	0 (0)	0 (0)	0 (0)	0 (0)
(95% CI)	(0.0–1.6)	(0.0–4.7)	(0.0–2.1)	(0.0–6.1)	(0.0–7.1)	(0.0–19.5)
2nd Dose—*n*	215	69	168	54	47	15
With one or more AEs—*n* (%)	10 (4.7)	5 (7.2)	6 (3.6)	3 (5.6)	4 (8.5)	2 (13.3)
(95% CI)	(2.3–8.4)	(2.4–16.1)	(1.3–7.6)	(1.2–15.4)	(2.4–20.4)	(1.7–40.5)
Mild (Grade 1)—*n* (%)	5 (2.3)	4 (5.8)	3 (1.8)	2 (3.7)	2 (4.3)	2 (13.3)
Moderate (Grade 2)—*n* (%)	5 (2.3)	0 (0)	3 (1.8)	0 (0)	2 (4.3)	0 (0)
Severe (Grade 3)—*n* (%)	0 (0)	1 (1.4)	0 (0)	1 (1.9)	0 (0)	0 (0)
Potentially life-threatening (Grade 4)—*n* (%)	0 (0)	0 (0)	0 (0)	0 (0)	0 (0)	0 (0)
With vaccine-related AEs—*n* (%)	0 (0)	0 (0)	0 (0)	0 (0)	0 (0)	0 (0)
(95% CI)	(0.0–1.7)	(0.0–5.2)	(0.0–2.2)	(0.0–6.6)	(0.0–7.5)	(0.0–21.8)
Serious adverse events (SAEs)						
1st Dose—*n*	224	76	174	59	50	17
With one or more SAEs—*n* (%)	0 (0)	1 (1.3)	0 (0)	1 (1.7)	0 (0)	0 (0)
(95% CI)	(0.0–1.6)	(0.0–7.1)	(0.0–2.1)	(0.0–9.1)	(0.0–7.1)	(0.0–19.5)
With vaccine-related SAEs—*n* (%)	0 (0)	0 (0)	0 (0)	0 (0)	0 (0)	0 (0)
(95% CI)	(0.0–1.6)	(0.0–4.7)	(0.0–2.1)	(0.0–6.1)	(0.0–7.1)	(0.0–19.5)
SAEs leading to death—*n* (%)	0 (0)	0 (0)	0 (0)	0 (0)	0 (0)	0 (0)
(95% CI)	(0.0–1.6)	(0.0–4.7)	(0.0–2.1)	(0.0–6.1)	(0.0–7.1)	(0.0–19.5)
With vaccine-related SAEs leading to death—*n* (%)	0 (0)	0 (0)	0 (0)	0 (0)	0 (0)	0 (0)
(95% CI)	(0.0–1.6)	(0.0–4.7)	(0.0–2.1)	(0.0–6.1)	(0.0–7.1)	(0.0–19.5)
2nd Dose—*n*	215	69	168	54	47	15
With one or more SAEs—*n* (%)	0 (0)	1 (1.4)	0 (0)	1 (1.9)	0 (0)	0 (0)
(95% CI)	(0.0–1.7)	(0.0–7.8)	(0.0–2.2)	(0.0–9.9)	(0.0–7.5)	(0.0–21.8)
With vaccine-related SAEs—*n* (%)	0 (0)	0 (0)	0 (0)	0 (0)	0 (0)	0 (0)
(95% CI)	(0.0–1.7)	(0.0–5.2)	(0.0–2.2)	(0.0–6.6)	(0.0–7.5)	(0.0–21.8)
SAEs leading to death—*n* (%)	0 (0)	0 (0)	0 (0)	0 (0)	0 (0)	0 (0)
(95% CI)	(0.0–1.7)	(0.0–5.2)	(0.0–2.2)	(0.0–6.6)	(0.0–7.5)	(0.0–21.8)
With vaccine-related SAEs leading to death—*n* (%)	0 (0)	0 (0)	0 (0)	0 (0)	0 (0)	0 (0)
(95% CI)	(0.0–1.7)	(0.0–5.2)	(0.0–2.2)	(0.0–6.6)	(0.0–7.5)	(0.0–21.8)
Medically attended adverse events (MAAEs)						
1st Dose—*n*	224	76	174	59	50	17
With one or more MAAEs—*n* (%)	17 (7.6)	8 (10.5)	13 (7.5)	6 (10.2)	4 (8.0)	2 (11.8)
(95% CI)	(4.5–11.9)	(4.7–19.7)	(4.0–12.4)	(3.8–20.8)	(2.2–19.2)	(1.5–36.4)
With vaccine-related MAAEs—*n* (%)	0 (0)	0 (0)	0 (0)	0 (0)	0 (0)	0 (0)
(95% CI)	(0.0–1.6)	(0.0–4.7)	(0.0–2.1)	(0.0–6.1)	(0.0–7.1)	(0.0–19.5)
2nd Dose—*n*	215	69	168	54	47	15
With one or more MAAEs—*n* (%)	3 (1.4)	5 (7.2)	0 (0)	3 (5.6)	3 (6.4)	2 (13.3)
(95% CI)	(0.3–4.0)	(2.4–16.1)	(0.0–2.2)	(1.2–15.4)	(1.3–17.5)	(1.7–40.5)
With vaccine-related MAAEs—*n* (%)	0 (0)	0 (0)	0 (0)	0 (0)	0 (0)	0 (0)
(95% CI)	(0.0–1.7)	(0.0–5.2)	(0.0–2.2)	(0.0–6.6)	(0.0–7.5)	(0.0–21.8)
Adverse events of special interest (AESIs)						
1st Dose—*n*	224	76	174	59	50	17
With one or more AESIs—*n* (%)	0 (0)	0 (0)	0 (0)	0 (0)	0 (0)	0 (0)
(95% CI)	(0.0–1.6)	(0.0–4.7)	(0.0–2.1)	(0.0–6.1)	(0.0–7.1)	(0.0–19.5)
With vaccine-related AESIs—*n* (%)	0 (0)	0 (0)	0 (0)	0 (0)	0 (0)	0 (0)
(95% CI)	(0.0–1.6)	(0.0–4.7)	(0.0–2.1)	(0.0–6.1)	(0.0–7.1)	(0.0–19.5)
2nd Dose—*n*	215	69	168	54	47	15
With one or more AESIs—*n* (%)	0 (0)	0 (0)	0 (0)	0 (0)	0 (0)	0 (0)
(95% CI)	(0.0–1.7)	(0.0–5.2)	(0.0–2.2)	(0.0–6.6)	(0.0–7.5)	(0.0–21.8)
With vaccine-related AESIs—*n* (%)	0 (0)	0 (0)	0 (0)	0 (0)	0 (0)	0 (0)
(95% CI)	(0.0–1.7)	(0.0–5.2)	(0.0–2.2)	(0.0–6.6)	(0.0–7.5)	(0.0–21.8)
Serious adverse events (SAEs) throughout the study						
With one or more SAEs—*n* (%)	5 (2.2)	2 (2.6)	3 (1.7)	2 (3.4)	2 (4.0)	0 (0)
Severe (Grade 3)—*n* (%)	2 (0.9)	2 (2.6)	1 (0.6)	2 (3.4)	1 (2.0)	0 (0)
Potentially life-threatening (Grade 4)—*n* (%)	0 (0)	0 (0)	0 (0)	0 (0)	0 (0)	0 (0)
Serious adverse events leading to death (Grade 5)—*n* (%)	3 (1.3)	0 (0)	2 (1.1)	0 (0)	1 (2.0)	0 (0)
With vaccine-related SAEs—*n* (%)	0 (0)	0 (0)	0 (0)	0 (0)	0 (0)	0 (0)
Medically attended adverse events (MAAEs) throughout the study						
With MAAEs—*n* (%)	38 (17.0)	17 (22.4)	27 (15.5)	12 (20.3)	11 (22.0)	5 (29.4)
Mild (Grade 1)—*n* (%)	28 (12.5)	13 (17.1)	23 (13.2)	9 (15.3)	5 (10.0)	4 (23.5)
Moderate (Grade 2)—*n* (%)	6 (2.7)	1 (1.3)	2 (1.1)	1 (1.7)	4 (8.0)	0 (0)
Severe (Grade 3)—*n* (%)	3 (1.3)	3 (3.9)	2 (1.1)	2 (3.4)	1 (2.0)	1 (5.9)
Potentially life-threatening (Grade 4)—*n* (%)	0 (0)	0 (0)	0 (0)	0 (0)	0 (0)	0 (0)
Fatal (Grade 5)—*n* (%)	1 (0.4)	0 (0)	0 (0)	0 (0)	1 (2.0)	0 (0)
With vaccine-related MAAEs—*n* (%)	0 (0)	0 (0)	0 (0)	0 (0)	0 (0)	0 (0)
Adverse events of special interest (AESIs) throughout the study						
With one or more AESIs—*n* (%)	0 (0)	0 (0)	0 (0)	0 (0)	0 (0)	0 (0)
With vaccine-related AESIs—*n* (%)	0 (0)	0 (0)	0 (0)	0 (0)	0 (0)	0 (0)

Abbreviations: SAE, serious adverse event; MAAE, medically attended adverse event; AESI, adverse event of special interest; CI, confidence interval. Note: Data are *n* (%). For dose-specific summaries, denominators are the number of participants vaccinated at that dose. For entire-study-period summaries, denominators are the number of participants who received any dose. Relatedness and seriousness were assessed by investigators; 95% CIs were calculated using the Clopper–Pearson exact method.

**Table 4:** Geometric mean titer or concentration ratios for 50% neutralizing antibody titers measured by pseudovirus neutralization assay and anti-spike immunoglobulin G concentrations measured by enzyme-linked immunosorbent assay at baseline, 28 days after the first dose (D29), 14 days after the second dose (D43), and 6 months after the second dose (D197), overall and by age stratum (per-protocol population).

	Overall	18–59 yrs	≥60 yrs
	HXP-GPOVac/BNT162b2	HXP-GPOVac/BNT162b2	HXP-GPOVac/BNT162b2
Baseline (D1)			
NT_50_ GMT ratio (95% CI)	0.87 (0.73–1.04)	0.79 (0.64–0.97)	1.22 (0.83–1.81)
Anti-spike IgG GMC ratio (95% CI)	0.89 (0.63–1.27)	0.73 (0.48–1.10)	1.76 (0.95–3.27)
28 days after the 1st vaccination (D29)			
NT_50_ GMT ratio (95% CI)	0.97 (0.53–1.76)	0.71 (0.37–1.39)	2.92 (0.80–10.60)
Anti-spike IgG GMC ratio (95% CI)	0.18 (0.10–0.30)	0.14 (0.08–0.26)	0.41 (0.13–1.28)
14 days after the 2nd vaccination (D43)			
NT_50_ GMT ratio (95% CI)	0.51 (0.39–0.67)	0.47 (0.35–0.63)	0.68 (0.37–1.24)
Anti-spike IgG GMC ratio (95% CI)	0.15 (0.12–0.19)	0.14 (0.11–0.19)	0.17 (0.09–0.30)
6 months after the 2nd vaccination (D197)			
NT_50_ GMT ratio (95% CI)	0.45 (0.29–0.69)	0.37 (0.23–0.61)	0.84 (0.31–2.31)
Anti-spike IgG GMC ratio (95% CI)	0.26 (0.17–0.38)	0.22 (0.15–0.35)	0.40 (0.16–1.00)

Abbreviations: NT_50_, 50% neutralization titer; GMT, geometric mean titer; GMC, geometric mean concentration; CI, confidence interval; D#, visit day (e.g., D1 = visit day 1, D29 = visit day 29, etc.). Note: Ratios are group geometric mean ratios calculated as HXP-GPOVac/BNT162b2; 95% CIs are calculated from log-transformed data.

## Data Availability

The data presented in this study are available on request from the corresponding author due to participant privacy and ethical restrictions.

## Supplementary Materials

The following supporting information can be downloaded at: https://www.mdpi.com/article/10.3390/vaccines14060481/s1. Table S1: Incidence of solicited local and systemic adverse events during the first 7 days after each vaccination, by randomization arm and age stratum; Table S2: Solicited adverse event severity grading criteria; Table S3: Geometric mean titers or concentrations, geometric mean fold rises, and seroconversion rates for neutralizing antibody titers and anti-spike IgG concentrations; Table S4: Cell-mediated immune responses to SARS-CoV-2 spike; Table S5: Clinical laboratory hematology results by treatment group; Table S6: Clinical laboratory serum chemistry results by treatment group.
