# Supplementary material for: Safety and Immunogenicity of a Locally Produced Inactivated NDV-HXP-S COVID-19 Vaccine (HXP-GPOVac) Compared with BNT162b2: A Phase II Randomized, Controlled, Double-Blind Noninferiority Trial in Thai Adults
Source: Vaccines (Basel). 2026 May 28;14(6):481. doi: 10.3390/vaccines14060481 (PMC13307917; doi:10.3390/vaccines14060481)
Supplement: Supplementary file 1 [file vaccines-14-00481-s001.zip › vaccines-4316209-supplementary.pdf]

Supplementary Materials

Table S1 – Incidence of solicited local and systemic adverse events during the first 7 days after each vaccination, by randomization arm and age stratum (safety population).

	Overall		18 – 59 yrs		≥ 60 yrs	
	HXP-GPOVac (N = 224)	BNT162b2 (N = 76)	HXP-GPOVac (N = 174)	BNT162b2 (N = 59)	HXP-GPOVac (N = 50)	BNT162b2 (N = 17)
Solicited local adverse events						
1st Dose – n	224	76	174	59	50	17
Any local reaction – n (%)	53 (23.7)	34 (44.7)	46 (26.4)	28 (47.5)	7 (14.0)	6 (35.3)
(95% CI)	(18.3–29.8)	(33.3–56.6)	(20.1–33.6)	(34.3–60.9)	(5.8–26.7)	(14.2–61.7)
2nd Dose – n	214	69	167	54	47	15
Any local reaction – n (%)	38 (17.8)	17 (24.6)	32 (19.2)	13 (24.1)	6 (12.8)	4 (26.7)
(95% CI)	(12.9–23.5)	(15.1–36.5)	(13.5–26.0)	(13.5–37.6)	(4.8–25.7)	(7.8–55.1)
Pain or Tenderness						
1st Dose – n	224	76	174	59	50	17
With one or more – n (%)	51 (22.8)	34 (44.7)	44 (25.3)	28 (47.5)	7 (14.0)	6 (35.3)
(95% CI)	(17.4–28.8)	(33.3–56.6)	(19.0–32.4)	(34.3–60.9)	(5.8–26.7)	(14.2–61.7)
Mild (Grade 1) – n (%)	48 (21.4)	31 (40.8)	43 (24.7)	25 (42.4)	5 (10.0)	6 (35.3)
(95% CI)	(16.2–27.4)	(29.6–52.7)	(18.5–31.8)	(29.6–55.9)	(3.3–21.8)	(14.2–61.7)
Moderate (Grade 2) – n (%)	3 (1.3)	3 (3.9)	1 (0.6)	3 (5.1)	2 (4.0)	0 (0)
(95% CI)	(0.3–3.9)	(0.8–11.1)	(0.0–3.2)	(1.1–14.1)	(0.5–13.7)	(0.0–19.5)
Severe (Grade 3) – n (%)	0 (0)	0 (0)	0 (0)	0 (0)	0 (0)	0 (0)
(95% CI)	(0.0–1.6)	(0.0–4.7)	(0.0–2.1)	(0.0–6.1)	(0.0–7.1)	(0.0–19.5)
Potentially life-threatening (Grade 4) – n (%)	0 (0)	0 (0)	0 (0)	0 (0)	0 (0)	0 (0)
(95% CI)	(0.0–1.6)	(0.0–4.7)	(0.0–2.1)	(0.0–6.1)	(0.0–7.1)	(0.0–19.5)
2nd Dose – n	214	69	167	54	47	15
With one or more – n (%)	38 (17.8)	17 (24.6)	32 (19.2)	13 (24.1)	6 (12.8)	4 (26.7)
(95% CI)	(12.9–23.5)	(15.1–36.5)	(13.5–26.0)	(13.5–37.6)	(4.8–25.7)	(7.8–55.1)
Mild (Grade 1) – n (%)	31 (14.5)	12 (17.4)	26 (15.6)	8 (14.8)	5 (10.6)	4 (26.7)
(95% CI)	(10.1–19.9)	(9.3–28.4)	(10.4–22.0)	(6.6–27.1)	(3.5–23.1)	(7.8–55.1)
Moderate (Grade 2) – n (%)	7 (3.3)	5 (7.2)	6 (3.6)	5 (9.3)	1 (2.1)	0 (0)
(95% CI)	(1.3–6.6)	(2.4–16.1)	(1.3–7.7)	(3.1–20.3)	(0.1–11.3)	(0.0–21.8)
Severe (Grade 3) – n (%)	0 (0)	0 (0)	0 (0)	0 (0)	0 (0)	0 (0)
(95% CI)	(0.0–1.7)	(0.0–5.2)	(0.0–2.2)	(0.0–6.6)	(0.0–7.5)	(0.0–21.8)
Potentially life-threatening (Grade 4) – n (%)	0 (0)	0 (0)	0 (0)	0 (0)	0 (0)	0 (0)
(95% CI)	(0.0–1.7)	(0.0–5.2)	(0.0–2.2)	(0.0–6.6)	(0.0–7.5)	(0.0–21.8)
Swelling or Induration						

	Overall		18 – 59 yrs		≥ 60 yrs	
	HXP-GPOVac (N = 224)	BNT162b2 (N = 76)	HXP-GPOVac (N = 174)	BNT162b2 (N = 59)	HXP-GPOVac (N = 50)	BNT162b2 (N = 17)
1st Dose – n	224	76	174	59	50	17
With one or more – n (%)	2 (0.9)	0 (0)	2 (1.1)	0 (0)	0 (0)	0 (0)
(95% CI)	(0.1–3.2)	(0.0–4.7)	(0.1–4.1)	(0.0–6.1)	(0.0–7.1)	(0.0–19.5)
Mild (Grade 1) – n (%)	2 (0.9)	0 (0)	2 (1.1)	0 (0)	0 (0)	0 (0)
(95% CI)	(0.1–3.2)	(0.0–4.7)	(0.1–4.1)	(0.0–6.1)	(0.0–7.1)	(0.0–19.5)
Moderate (Grade 2) – n (%)	0 (0)	0 (0)	0 (0)	0 (0)	0 (0)	0 (0)
(95% CI)	(0.0–1.6)	(0.0–4.7)	(0.0–2.1)	(0.0–6.1)	(0.0–7.1)	(0.0–19.5)
Severe (Grade 3) – n (%)	0 (0)	0 (0)	0 (0)	0 (0)	0 (0)	0 (0)
(95% CI)	(0.0–1.6)	(0.0–4.7)	(0.0–2.1)	(0.0–6.1)	(0.0–7.1)	(0.0–19.5)
Potentially life-threatening (Grade 4) – n (%)	0 (0)	0 (0)	0 (0)	0 (0)	0 (0)	0 (0)
(95% CI)	(0.0–1.6)	(0.0–4.7)	(0.0–2.1)	(0.0–6.1)	(0.0–7.1)	(0.0–19.5)
2nd Dose – n	214	69	167	54	47	15
With one or more – n (%)	0 (0)	0 (0)	0 (0)	0 (0)	0 (0)	0 (0)
(95% CI)	(0.0–1.7)	(0.0–5.2)	(0.0–2.2)	(0.0–6.6)	(0.0–7.5)	(0.0–21.8)
Mild (Grade 1) – n (%)	0 (0)	0 (0)	0 (0)	0 (0)	0 (0)	0 (0)
(95% CI)	(0.0–1.7)	(0.0–5.2)	(0.0–2.2)	(0.0–6.6)	(0.0–7.5)	(0.0–21.8)
Moderate (Grade 2) – n (%)	0 (0)	0 (0)	0 (0)	0 (0)	0 (0)	0 (0)
(95% CI)	(0.0–1.7)	(0.0–5.2)	(0.0–2.2)	(0.0–6.6)	(0.0–7.5)	(0.0–21.8)
Severe (Grade 3) – n (%)	0 (0)	0 (0)	0 (0)	0 (0)	0 (0)	0 (0)
(95% CI)	(0.0–1.7)	(0.0–5.2)	(0.0–2.2)	(0.0–6.6)	(0.0–7.5)	(0.0–21.8)
Potentially life-threatening (Grade 4) – n (%)	0 (0)	0 (0)	0 (0)	0 (0)	0 (0)	0 (0)
(95% CI)	(0.0–1.7)	(0.0–5.2)	(0.0–2.2)	(0.0–6.6)	(0.0–7.5)	(0.0–21.8)
Erythema						
1st Dose – n	224	76	174	59	50	17
With one or more – n (%)	0 (0)	2 (2.6)	0 (0)	2 (3.4)	0 (0)	0 (0)
(95% CI)	(0.0–1.6)	(0.3–9.2)	(0.0–2.1)	(0.4–11.7)	(0.0–7.1)	(0.0–19.5)
Mild (Grade 1) – n (%)	0 (0)	1 (1.3)	0 (0)	1 (1.7)	0 (0)	0 (0)
(95% CI)	(0.0–1.6)	(0.0–7.1)	(0.0–2.1)	(0.0–9.1)	(0.0–7.1)	(0.0–19.5)
Moderate (Grade 2) – n (%)	0 (0)	1 (1.3)	0 (0)	1 (1.7)	0 (0)	0 (0)
(95% CI)	(0.0–1.6)	(0.0–7.1)	(0.0–2.1)	(0.0–9.1)	(0.0–7.1)	(0.0–19.5)
Severe (Grade 3) – n (%)	0 (0)	0 (0)	0 (0)	0 (0)	0 (0)	0 (0)
(95% CI)	(0.0–1.6)	(0.0–4.7)	(0.0–2.1)	(0.0–6.1)	(0.0–7.1)	(0.0–19.5)
Potentially life-threatening (Grade 4) – n (%)	0 (0)	0 (0)	0 (0)	0 (0)	0 (0)	0 (0)
(95% CI)	(0.0–1.6)	(0.0–4.7)	(0.0–2.1)	(0.0–6.1)	(0.0–7.1)	(0.0–19.5)
2nd Dose – n	214	69	167	54	47	15
With one or more – n (%)	0 (0)	0 (0)	0 (0)	0 (0)	0 (0)	0 (0)
(95% CI)	(0.0–1.7)	(0.0–5.2)	(0.0–2.2)	(0.0–6.6)	(0.0–7.5)	(0.0–21.8)

	Overall		18 – 59 yrs		≥ 60 yrs	
	HXP-GPOVac (N = 224)	BNT162b2 (N = 76)	HXP-GPOVac (N = 174)	BNT162b2 (N = 59)	HXP-GPOVac (N = 50)	BNT162b2 (N = 17)
Mild (Grade 1) – n (%)	0 (0)	0 (0)	0 (0)	0 (0)	0 (0)	0 (0)
(95% CI)	(0.0–1.7)	(0.0–5.2)	(0.0–2.2)	(0.0–6.6)	(0.0–7.5)	(0.0–21.8)
Moderate (Grade 2) – n (%)	0 (0)	0 (0)	0 (0)	0 (0)	0 (0)	0 (0)
(95% CI)	(0.0–1.7)	(0.0–5.2)	(0.0–2.2)	(0.0–6.6)	(0.0–7.5)	(0.0–21.8)
Severe (Grade 3) – n (%)	0 (0)	0 (0)	0 (0)	0 (0)	0 (0)	0 (0)
(95% CI)	(0.0–1.7)	(0.0–5.2)	(0.0–2.2)	(0.0–6.6)	(0.0–7.5)	(0.0–21.8)
Potentially life-threatening (Grade 4) – n (%)	0 (0)	0 (0)	0 (0)	0 (0)	0 (0)	0 (0)
(95% CI)	(0.0–1.7)	(0.0–5.2)	(0.0–2.2)	(0.0–6.6)	(0.0–7.5)	(0.0–21.8)
Solicited systemic adverse events						
1st Dose – n	224	76	174	59	50	17
Any local reaction – n (%)	67 (29.9)	28 (36.8)	62 (35.6)	25 (42.4)	5 (10.0)	3 (17.6)
(95% CI)	(24.0–36.4)	(26.1–48.7)	(28.5–43.2)	(29.6–55.9)	(3.3–21.8)	(3.8–43.4)
2nd Dose – n	214	69	167	54	47	15
Any local reaction – n (%)	43 (20.1)	16 (23.2)	37 (22.2)	13 (24.1)	6 (12.8)	3 (20)
(95% CI)	(14.9–26.1)	(13.9–34.9)	(16.1–29.2)	(13.5–37.6)	(4.8–25.7)	(4.3–48.1)
Fever (≥ 38 °C)						
1st Dose – n	224	76	174	59	50	17
With one or more – n (%)	0 (0)	0 (0)	0 (0)	0 (0)	0 (0)	0 (0)
(95% CI)	(0.0–1.6)	(0.0–4.7)	(0.0–2.1)	(0.0–6.1)	(0.0–7.1)	(0.0–19.5)
Mild (Grade 1) – n (%)	0 (0)	0 (0)	0 (0)	0 (0)	0 (0)	0 (0)
(95% CI)	(0.0–1.6)	(0.0–4.7)	(0.0–2.1)	(0.0–6.1)	(0.0–7.1)	(0.0–19.5)
Moderate (Grade 2) – n (%)	0 (0)	0 (0)	0 (0)	0 (0)	0 (0)	0 (0)
(95% CI)	(0.0–1.6)	(0.0–4.7)	(0.0–2.1)	(0.0–6.1)	(0.0–7.1)	(0.0–19.5)
Severe (Grade 3) – n (%)	0 (0)	0 (0)	0 (0)	0 (0)	0 (0)	0 (0)
(95% CI)	(0.0–1.6)	(0.0–4.7)	(0.0–2.1)	(0.0–6.1)	(0.0–7.1)	(0.0–19.5)
Potentially life-threatening (Grade 4) – n (%)	0 (0)	0 (0)	0 (0)	0 (0)	0 (0)	0 (0)
(95% CI)	(0.0–1.6)	(0.0–4.7)	(0.0–2.1)	(0.0–6.1)	(0.0–7.1)	(0.0–19.5)
2nd Dose – n	214	69	67	54	47	15
With one or more – n (%)	0 (0)	0 (0)	0 (0)	0 (0)	0 (0)	0 (0)
(95% CI)	(0.0–1.7)	(0.0–5.2)	(0.0–2.2)	(0.0–6.6)	(0.0–7.5)	(0.0–21.8)
Mild (Grade 1) – n (%)	0 (0)	0 (0)	0 (0)	0 (0)	0 (0)	0 (0)
(95% CI)	(0.0–1.7)	(0.0–5.2)	(0.0–2.2)	(0.0–6.6)	(0.0–7.5)	(0.0–21.8)
Moderate (Grade 2) – n (%)	0 (0)	0 (0)	0 (0)	0 (0)	0 (0)	0 (0)
(95% CI)	(0.0–1.7)	(0.0–5.2)	(0.0–2.2)	(0.0–6.6)	(0.0–7.5)	(0.0–21.8)
Severe (Grade 3) – n (%)	0 (0)	0 (0)	0 (0)	0 (0)	0 (0)	0 (0)
(95% CI)	(0.0–1.7)	(0.0–5.2)	(0.0–2.2)	(0.0–6.6)	(0.0–7.5)	(0.0–21.8)

	Overall		18 – 59 yrs		≥ 60 yrs	
	HXP-GPOVac (N = 224)	BNT162b2 (N = 76)	HXP-GPOVac (N = 174)	BNT162b2 (N = 59)	HXP-GPOVac (N = 50)	BNT162b2 (N = 17)
Potentially life-threatening (Grade 4) – n (%)	0 (0)	0 (0)	0 (0)	0 (0)	0 (0)	0 (0)
(95% CI)	(0.0–1.7)	(0.0–5.2)	(0.0–2.2)	(0.0–6.6)	(0.0–7.5)	(0.0–21.8)
Headache						
1st Dose – n	224	76	174	59	50	17
With one or more – n (%)	33 (14.7)	16 (21.1)	31 (17.8)	14 (23.7)	2 (4.0)	2 (11.8)
(95% CI)	(10.4–20.1)	(12.5–31.9)	(12.4–24.3)	(13.6–36.6)	(0.5–13.7)	(1.5–36.4)
Mild (Grade 1) – n (%)	30 (13.4)	14 (18.4)	28 (16.1)	12 (20.3)	2 (4.0)	2 (11.8)
(95% CI)	(9.2–18.6)	(10.5–29.0)	(11.0–22.4)	(11.0–32.8)	(0.5–13.7)	(1.5–36.4)
Moderate (Grade 2) – n (%)	3 (1.3)	2 (2.6)	3 (1.7)	2 (3.4)	0 (0)	0 (0)
(95% CI)	(0.3–3.9)	(0.3–9.2)	(0.4–5.0)	(0.4–11.7)	(0.0–7.1)	(0.0–19.5)
Severe (Grade 3) – n (%)	0 (0)	0 (0)	0 (0)	0 (0)	0 (0)	0 (0)
(95% CI)	(0.0–1.6)	(0.0–4.7)	(0.0–2.1)	(0.0–6.1)	(0.0–7.1)	(0.0–19.5)
Potentially life-threatening (Grade 4) – n (%)	0 (0)	0 (0)	0 (0)	0 (0)	0 (0)	0 (0)
(95% CI)	(0.0–1.6)	(0.0–4.7)	(0.0–2.1)	(0.0–6.1)	(0.0–7.1)	(0.0–19.5)
2nd Dose – n	214	69	167	54	47	15
With one or more – n (%)	23 (10.7)	7 (10.1)	20 (12.0)	5 (9.3)	3 (6.4)	2 (13.3)
(95% CI)	(6.9–15.7)	(4.2–19.8)	(7.5–17.9)	(3.1–20.3)	(1.3–17.5)	(1.7–40.5)
Mild (Grade 1) – n (%)	17 (7.9)	5 (7.2)	15 (9.0)	3 (5.6)	2 (4.3)	2 (13.3)
(95% CI)	(4.7–12.4)	(2.4–16.1)	(5.1–14.4)	(1.2–15.4)	(0.5–14.5)	(1.7–40.5)
Moderate (Grade 2) – n (%)	6 (2.8)	2 (2.9)	5 (3.0)	2 (3.7)	1 (2.1)	0 (0)
(95% CI)	(1.0–6.0)	(0.4–10.1)	(1.0–6.8)	(0.5–12.7)	(0.1–11.3)	(0.0–21.8)
Severe (Grade 3) – n (%)	0 (0)	0 (0)	0 (0)	0 (0)	0 (0)	0 (0)
(95% CI)	(0.0–1.7)	(0.0–5.2)	(0.0–2.2)	(0.0–6.6)	(0.0–7.5)	(0.0–21.8)
Potentially life-threatening (Grade 4) – n (%)	0 (0)	0 (0)	0 (0)	0 (0)	0 (0)	0 (0)
(95% CI)	(0.0–1.7)	(0.0–5.2)	(0.0–2.2)	(0.0–6.6)	(0.0–7.5)	(0.0–21.8)
Fatigue or Malaise						
1st Dose – n	224	76	174	59	50	17
With one or more – n (%)	38 (17.0)	14 (18.4)	35 (20.1)	14 (23.7)	3 (6.0)	0 (0)
(95% CI)	(12.3–22.5)	(10.5–29.0)	(14.4–26.8)	(13.6–36.6)	(1.3–16.5)	(0.0–19.5)
Mild (Grade 1) – n (%)	33 (14.7)	11 (14.5)	31 (17.8)	11 (18.6)	2 (4.0)	0 (0)
(95% CI)	(10.4–20.1)	(7.5–24.4)	(12.4–24.3)	(9.7–30.9)	(0.5–13.7)	(0.0–19.5)
Moderate (Grade 2) – n (%)	5 (2.2)	3 (3.9)	4 (2.3)	3 (5.1)	1 (2.0)	0 (0)
(95% CI)	(0.7–5.1)	(0.8–11.1)	(0.6–5.8)	(1.1–14.1)	(0.1–10.6)	(0.0–19.5)
Severe (Grade 3) – n (%)	0 (0)	0 (0)	0 (0)	0 (0)	0 (0)	0 (0)
(95% CI)	(0.0–1.6)	(0.0–4.7)	(0.0–2.1)	(0.0–6.1)	(0.0–7.1)	(0.0–19.5)
Potentially life-threatening (Grade 4) – n (%)	0 (0)	0 (0)	0 (0)	0 (0)	0 (0)	0 (0)

	Overall		18 – 59 yrs		≥ 60 yrs	
	HXP-GPOVac (N = 224)	BNT162b2 (N = 76)	HXP-GPOVac (N = 174)	BNT162b2 (N = 59)	HXP-GPOVac (N = 50)	BNT162b2 (N = 17)
(95% CI)	(0.0–1.6)	(0.0–4.7)	(0.0–2.1)	(0.0–6.1)	(0.0–7.1)	(0.0–19.5)
2nd Dose – n	214	69	167	54	47	15
With one or more – n (%)	29 (13.6)	9 (13.0)	25 (15.0)	7 (13.0)	4 (8.5)	2 (13.3)
(95% CI)	(9.3–18.9)	(6.1–23.3)	(9.9–21.3)	(5.4–24.9)	(2.4–20.4)	(1.7–40.5)
Mild (Grade 1) – n (%)	25 (11.7)	7 (10.10)	21 (12.6)	5 (9.3)	4 (8.5)	2 (13.3)
(95% CI)	(7.7–16.8)	(4.2–19.8)	(8.0–18.6)	(3.1–20.3)	(2.4–20.4)	(1.7–40.5)
Moderate (Grade 2) – n (%)	4 (1.9)	2 (2.9)	4 (2.4)	2 (3.7)	0 (0)	0 (0)
(95% CI)	(0.5–4.7)	(0.4–10.1)	(0.7–6.0)	(0.5–12.7)	(0.0–7.5)	(0.0–21.8)
Severe (Grade 3) – n (%)	0 (0)	0 (0)	0 (0)	0 (0)	0 (0)	0 (0)
(95% CI)	(0.0–1.7)	(0.0–5.2)	(0.0–2.2)	(0.0–6.6)	(0.0–7.5)	(0.0–21.8)
Potentially life-threatening (Grade 4) – n (%)	0 (0)	0 (0)	0 (0)	0 (0)	0 (0)	0 (0)
(95% CI)	(0.0–1.7)	(0.0–5.2)	(0.0–2.2)	(0.0–6.6)	(0.0–7.5)	(0.0–21.8)
Myalgia						
1st Dose – n	224	76	174	59	50	17
With one or more – n (%)	47 (21.0)	19 (25.0)	43 (24.7)	18 (30.5)	4 (8.0)	1 (5.9)
(95% CI)	(15.8–26.9)	(15.8–36.3)	(18.5–31.8)	(19.2–43.9)	(2.2–19.2)	(0.1–28.7)
Mild (Grade 1) – n (%)	43 (19.2)	14 (18.4)	40 (23.0)	13 (22.0)	3 (6.0)	1 (5.9)
(95% CI)	(14.3–25.0)	(10.5–29.0)	(17.0–30.0)	(12.3–34.7)	(1.3–16.5)	(0.1–28.7)
Moderate (Grade 2) – n (%)	4 (1.8)	5 (6.6)	3 (1.7)	5 (8.5)	1 (2.0)	0 (0)
(95% CI)	(0.5–4.5)	(2.2–14.7)	(0.4–5.0)	(2.8–18.7)	(0.1–10.6)	(0.0–19.5)
Severe (Grade 3) – n (%)	0 (0)	0 (0)	0 (0)	0 (0)	0 (0)	0 (0)
(95% CI)	(0.0–1.6)	(0.0–4.7)	(0.0–2.1)	(0.0–6.1)	(0.0–7.1)	(0.0–19.5)
Potentially life-threatening (Grade 4) – n (%)	0 (0)	0 (0)	0 (0)	0 (0)	0 (0)	0 (0)
(95% CI)	(0.0–1.6)	(0.0–4.7)	(0.0–2.1)	(0.0–6.1)	(0.0–7.1)	(0.0–19.5)
2nd Dose – n	214	69	167	54	47	15
With one or more – n (%)	31 (14.5)	13 (18.8)	28 (16.8)	12 (22.2)	3 (6.4)	1 (6.7)
(95% CI)	(10.1–19.9)	(10.4–30.1)	(11.4–23.3)	(12.0–35.6)	(1.3–17.5)	(0.2–31.9)
Mild (Grade 1) – n (%)	24 (11.2)	11 (15.9)	21 (12.6)	10 (18.5)	3 (6.4)	1 (6.7)
(95% CI)	(7.3–16.2)	(8.2–26.7)	(8.0–18.6)	(9.3–31.4)	(1.3–17.5)	(0.2–31.9)
Moderate (Grade 2) – n (%)	7 (3.3)	2 (2.9)	7 (4.2)	2 (3.7)	0 (0)	0 (0)
(95% CI)	(1.3–6.6)	(0.4–10.1)	(1.7–8.4)	(0.5–12.7)	(0.0–7.5)	(0.0–21.8)
Severe (Grade 3) – n (%)	0 (0)	0 (0)	0 (0)	0 (0)	0 (0)	0 (0)
(95% CI)	(0.0–1.7)	(0.0–5.2)	(0.0–2.2)	(0.0–6.6)	(0.0–7.5)	(0.0–21.8)
Potentially life-threatening (Grade 4) – n (%)	0 (0)	0 (0)	0 (0)	0 (0)	0 (0)	0 (0)
(95% CI)	(0.0–1.7)	(0.0–5.2)	(0.0–2.2)	(0.0–6.6)	(0.0–7.5)	(0.0–21.8)
Arthralgia						

	Overall		18 – 59 yrs		≥ 60 yrs	
	HXP-GPOVac (N = 224)	BNT162b2 (N = 76)	HXP-GPOVac (N = 174)	BNT162b2 (N = 59)	HXP-GPOVac (N = 50)	BNT162b2 (N = 17)
1st Dose – n	224	76	174	59	50	17
With one or more – n (%)	26 (11.6)	5 (6.6)	24 (13.8)	5 (8.5)	2 (4.0)	0 (0)
(95% CI)	(7.7–16.5)	(2.2–14.7)	(9.0–19.8)	(2.8–18.7)	(0.5–13.7)	(0.0–19.5)
Mild (Grade 1) – n (%)	24 (10.7)	3 (3.9)	22 (12.6)	3 (5.1)	2 (4.0)	0 (0)
(95% CI)	(7.0–15.5)	(0.8–11.1)	(8.1–18.5)	(1.1–14.1)	(0.5–13.7)	(0.0–19.5)
Moderate (Grade 2) – n (%)	2 (0.9)	2 (2.6)	2 (1.1)	2 (3.4)	0 (0)	0 (0)
(95% CI)	(0.1–3.2)	(0.3–9.2)	(0.1–4.1)	(0.4–11.7)	(0.0–7.1)	(0.0–19.5)
Severe (Grade 3) – n (%)	0 (0)	0 (0)	0 (0)	0 (0)	0 (0)	0 (0)
(95% CI)	(0.0–1.6)	(0.0–4.7)	(0.0–2.1)	(0.0–6.1)	(0.0–7.1)	(0.0–19.5)
Potentially life-threatening (Grade 4) – n (%)	0 (0)	0 (0)	0 (0)	0 (0)	0 (0)	0 (0)
(95% CI)	(0.0–1.6)	(0.0–4.7)	(0.0–2.1)	(0.0–6.1)	(0.0–7.1)	(0.0–19.5)
2nd Dose – n	214	69	167	54	47	15
With one or more – n (%)	22 (10.3)	3 (4.3)	20 (12.0)	2 (3.7)	2 (4.3)	1 (6.7)
(95% CI)	(6.6–15.2)	(0.9–12.2)	(7.5–17.9)	(0.5–12.7)	(0.5–14.5)	(0.2–31.9)
Mild (Grade 1) – n (%)	20 (9.3)	2 (2.9)	18 (10.8)	1 (1.9)	2 (4.3)	1 (6.7)
(95% CI)	(5.8–14.1)	(0.4–10.1)	(6.5–16.5)	(0.0–9.9)	(0.5–14.5)	(0.2–31.9)
Moderate (Grade 2) – n (%)	2 (0.9)	1 (1.4)	2 (1.2)	1 (1.9)	0 (0)	0 (0)
(95% CI)	(0.1–3.3)	(0.0–7.8)	(0.1–4.3)	(0.0–9.9)	(0.0–7.5)	(0.0–21.8)
Severe (Grade 3) – n (%)	0 (0)	0 (0	0 (0)	0 (0)	0 (0)	0 (0)
(95% CI)	(0.0–1.7)	(0.0–5.2)	(0.0–2.2)	(0.0–6.6)	(0.0–7.5)	(0.0–21.8)
Potentially life-threatening (Grade 4) – n (%)	0 (0)	0 (0)	0 (0)	0 (0)	0 (0)	0 (0)
(95% CI)	(0.0–1.7)	(0.0–5.2)	(0.0–2.2)	(0.0–6.6)	(0.0–7.5)	(0.0–21.8)
Nausea or Vomiting						
1st Dose – n	224	76	174	59	50	17
With one or more – n (%)	7 (3.1)	3 (3.9)	7 (4.0)	3 (5.1)	0 (0)	0 (0)
(95% CI)	(1.3–6.3)	(0.8–11.1)	(1.6–8.1)	(1.1–14.1)	(0.0–7.1)	(0.0–19.5)
Mild (Grade 1) – n (%)	5 (2.2)	2 (2.6)	5 (2.9)	2 (3.4)	0 (0)	0 (0)
(95% CI)	(0.7–5.1)	(0.3–9.2)	(0.9–6.6)	(0.4–11.7)	(0.0–7.1)	(0.0–19.5)
Moderate (Grade 2) – n (%)	2 (0.9)	1 (1.3)	2 (1.1)	1 (1.7)	0 (0)	0 (0)
(95% CI)	(0.1–3.2)	(0.0–7.1)	(0.1–4.1)	(0.0–9.1)	(0.0–7.1)	(0.0–19.5)
Severe (Grade 3) – n (%)	0 (0)	0 (0)	0 (0)	0 (0)	0 (0)	0 (0)
(95% CI)	(0.0–1.6)	(0.0–4.7)	(0.0–2.1)	(0.0–6.1)	(0.0–7.1)	(0.0–19.5)
Potentially life-threatening (Grade 4) – n (%)	0 (0)	0 (0)	0 (0)	0 (0)	0 (0)	0 (0)
(95% CI)	(0.0–1.6)	(0.0–4.7)	(0.0–2.1)	(0.0–6.1)	(0.0–7.1)	(0.0–19.5)
2nd Dose – n	214	69	167	54	47	15
With one or more – n (%)	5 (2.3)	1 (1.4)	5 (3.0)	1 (1.9)	0 (0)	0 (0)
(95% CI)	(0.8–5.4)	(0.0–7.8)	(1.0–6.8)	(0.0–9.9)	(0.0–7.5)	(0.0–21.8)

	Overall		18 – 59 yrs		≥ 60 yrs	
	HXP-GPOVac (N = 224)	BNT162b2 (N = 76)	HXP-GPOVac (N = 174)	BNT162b2 (N = 59)	HXP-GPOVac (N = 50)	BNT162b2 (N = 17)
Mild (Grade 1) – n (%)	5 (2.3)	1 (1.4)	5 (3.0)	1 (1.9)	0 (0)	0 (0)
(95% CI)	(0.8–5.4)	(0.0–7.8)	(1.0–6.8)	(0.0–9.9)	(0.0–7.5)	(0.0–21.8)
Moderate (Grade 2) – n (%)	0 (0)	0 (0)	0 (0)	0 (0)	0 (0)	0 (0)
(95% CI)	(0.0–1.7)	(0.0–5.2)	(0.0–2.2)	(0.0–6.6)	(0.0–7.5)	(0.0–21.8)
Severe (Grade 3) – n (%)	0 (0)	0 (0)	0 (0)	0 (0)	0 (0)	0 (0)
(95% CI)	(0.0–1.7)	(0.0–5.2)	(0.0–2.2)	(0.0–6.6)	(0.0–7.5)	(0.0–21.8)
Potentially life-threatening (Grade 4) – n (%)	0 (0)	0 (0)	0 (0)	0 (0)	0 (0)	0 (0)
(95% CI)	(0.0–1.7)	(0.0–5.2)	(0.0–2.2)	(0.0–6.6)	(0.0–7.5)	(0.0–21.8)

Abbreviations: AE, adverse event; CI, confidence interval.
Note: Data are n (%) of participants by maximum grade (Grade 1 = mild, 2 = moderate, 3 = severe, 4 = life-threatening). Percentages are by dose. 95% CIs are exact (Clopper–Pearson).

Table S2 – Solicited adverse event severity grading criteria

Adverse Event	Mild (Grade 1)	Moderate (Grade 2)	Severe (Grade 3)	Potentially Life-Threatening (Grade 4)
Systemic Illness				
Illness or clinical AE (as defined according to applicable regulations)	No or minimal interference with usual activities; no medical intervention/ therapy required	Marked limitation in ability to perform usual activities; medical intervention/ therapy required	Marked limitation in ability to perform usual activities; medical intervention/ therapy required	Inability to perform basic functions OR Medical or operative intervention indicated to prevent permanent impairment, persistent disability, or death
Local Reaction to Injectable Product				
Injection site pain (pain without touching) OR Tenderness (pain when area touched)	Pain/tenderness causing no or minimal limitation of use of limb	Pain or tenderness causing greater than minimal limitation of use of limb	Pain/tenderness causing inability to perform usual activities	Pain/tenderness causing inability to perform basic functions OR Hospitalization indicated
Injection site erythema or induration	2.5 to < 5 cm in diameter OR 6.25 to < 25 cm2 surface area	≥ 5 to < 10 cm in diameter OR ≥ 25 to < 100 cm2 surface area	≥ 10 cm in diameter OR ≥ 100 cm2 surface area OR Ulceration OR Secondary infection OR Phlebitis OR	Potentially life-threatening consequences (e.g., abscess, exfoliative dermatitis, necrosis involving dermis or deeper tissue)

Adverse Event	Mild (Grade 1)	Moderate (Grade 2)	Severe (Grade 3)	Potentially Life-Threatening (Grade 4)
			Sterile abscess OR Drainage	
Injection site pruritus	Itching localized to the injection site that is relieved spontaneously or in < 48 hours of treatment	Itching beyond the injection site that is not generalized OR Itching localized to the injection site requiring ≥ 48 hours treatment	Generalized itching causing inability to perform usual social & functional activities	N/A
Systemic (General)				
Acute systemic allergic reaction	Localized urticaria (wheals) with no medical intervention indicated	Localized urticaria with medical intervention indicated OR Mild angioedema with no medical intervention indicated	Generalized urticaria OR Angioedema with medical intervention indicated OR Symptomatic mild bronchospasm	Acute anaphylaxis OR Life-threatening bronchospasm OR laryngeal edema
Fever – °C	38.0 – 38.6	38.7 – 39.3	39.4 – 40.5	> 40.5
Myalgia (generalized)	Muscle pain causing no or minimal interference with usual social and functional activities	Muscle pain causing greater than minimal interference with usual social and functional activities	Muscle pain causing inability to perform usual social and functional activities	Disabling muscle pain causing inability to perform basic self-care functions
Headache	Symptoms causing no or minimal interference with usual social and functional activities	Symptoms causing greater than minimal interference with usual social and functional activities	Symptoms causing inability to perform usual social and functional activities	Symptoms causing inability to perform basic self-care functions OR Hospitalization indicated OR Headache with significant impairment of alertness or other neurologic function
Chills	Symptoms causing no or minimal interference with	Symptoms causing greater than minimal interference with	Symptoms causing inability to perform usual	N/A

Adverse Event	Mild (Grade 1)	Moderate (Grade 2)	Severe (Grade 3)	Potentially Life-Threatening (Grade 4)
	usual social and functional activities	usual social and functional activities	social and functional activities	
Fatigue	Symptoms causing no or minimal interference with usual social and functional activities	Symptoms causing greater than minimal interference with usual social and functional activities	Symptoms causing inability to perform usual social and functional activities	Incapacitating symptoms of fatigue or malaise causing inability to perform basic self- care functions
Malaise	No interference with activity	Some interference with activity	Prevents daily activity	Hospitalization
Arthralgia	No interference with activity	Some interference with activity	Prevents daily activity	Hospitalization
Nausea/Vomiting	No interference with activity	Some interference with activity	Prevents daily activity	Hospitalization
Tachycardia – beats per minute	101 – 115	116 – 130	> 130	ER visit or hospitalization for arrhythmia
Bradycardia – beats per minute	50 – 54	45 – 49	< 45	ER visit or hospitalization for arrhythmia
Respiratory Rate – breaths per minute	17 – 20	21 – 25	> 25	Intubation

Table S3 – Geometric mean titers or concentrations, geometric mean fold rises, and seroconversion rates for 50% neutralizing antibody titers measured by pseudovirus neutralization assay and anti-spike immunoglobulin G concentrations measured by enzyme-linked immunosorbent assay at baseline, 28 days after the first dose (D29), 14 days after the second dose (D43), and 6 months after the second dose (D197), overall and by age stratum (per-protocol population)

	Overall		18 – 59 yrs		≥ 60 yrs	
	HXP-GPOVac (N = 224)	BNT162b2 (N = 76)	HXP-GPOVac (N = 174)	BNT162b2 (N = 59)	HXP-GPOVac (N = 50)	BNT162b2 (N = 17)
Baseline (D1)						
PNA – n	224	75	174	58	50	17
NT_{50} GMT – Reciprocal dilution	5.62	6.45	5.49	6.95	6.11	5.00
(95% CI)	(5.20–6.07)	(5.20–7.99)	(5.09–5.92)	(5.27–9.16)	(4.86–7.69)	(–)
ELISA – n	224	74	174	57	50	17

	Overall		18 – 59 yrs		≥ 60 yrs	
	HXP-GPOVac (N = 224)	BNT162b2 (N = 76)	HXP-GPOVac (N = 174)	BNT162b2 (N = 59)	HXP-GPOVac (N = 50)	BNT162b2 (N = 17)
Anti-spike IgG GMC – BAU/mL	7.45	8.35	7.57	10.41	7.03	3.99
(95% CI)	(6.32–8.77)	(5.82–11.98)	(6.28–9.12)	(6.65–16.30)	(4.96–9.96)	(2.96–5.37)
28 days after the 1st vaccination (D29) – n						
PNA – n	215	69	168	54	47	15
NT_{50} GMT – Reciprocal dilution	65.52	67.80	74.30	104.40	41.79	14.33
(95% CI)	(48.60–88.33)	(40.42–113.72)	(53.28–103.63)	(58.91–185.02)	(21.14–82.63)	(5.90–34.82)
NT_{50} GMFR – fold	6.06	5.52	6.97	8.02	3.68	1.43
(95% CI)	(4.52–8.12)	(3.39–8.99)	(5.03–9.67)	(4.66–13.81)	(1.91–7.08)	(0.59–3.48)
NT_{50} SCR – n (%)	105 (48.8)	36 (52.2)	87 (51.8)	32 (59.3)	18 (38.3)	4 (26.7)
(95% CI)	(42.0–55.7)	(39.8–64.4)	(44.0–59.5)	(45.0–72.4)	(24.5–53.6)	(7.8–55.1)
ELISA – n	215	68	168	53	47	15
Anti-spike IgG GMC – BAU/mL	102.73	579.53	113.85	808.65	71.14	174.67
(95% CI)	(77.71–135.81)	(400.29–839.04)	(82.97–156.22)	(552.06–1184.50)	(38.92–130.05)	(77.52–393.58)
Anti-spike IgG GMFR – fold	9.11	43.29	9.83	50.53	6.94	25.05
(95% CI)	(7.29–11.37)	(32.17–58.24)	(7.62–12.66)	(36.52–69.92)	(4.34–11.09)	(12.36–50.77)
Anti-spike IgG SCR – n (%)	136 (63.3)	64 (94.1)	112 (66.7)	51 (96.2)	24 (51.1)	13 (86.7)
(95% CI)	(56.4–69.7)	(85.6–98.4)	(59.0–73.7)	(87.0–99.5)	(36.1–65.9)	(59.5–98.3)
14 days after the 2nd vaccination (D43)						
PNA – n	211	69	164	54	47	15
NT_{50} GMT – Reciprocal dilution	504.97	993.34	528.41	1126.79	431.01	630.98
(95% CI)	(439.79–579.80)	(806.11–1224.07)	(452.95–616.45)	(890.29–1426.12)	(314.10–591.45)	(415.48–958.26)
NT_{50} GMFR – fold	46.66	80.82	49.51	86.58	37.93	63.10
(95% CI)	(40.30–54.02)	(62.88–103.89)	(42.08–58.25)	(63.97–117.18)	(27.04–53.21)	(41.55–95.83)
NT_{50} SCR – n (%)	206 (97.6)	67 (97.1)	161 (98.2)	52 (96.3)	45 (95.7)	15 (100)
(95% CI)	(94.6–99.2)	(89.9–99.6)	(94.7–99.6)	(87.3–99.5)	(85.5–99.5)	(78.2–100.0)
ELISA – n	211	68	164	53	47	15
Anti-spike IgG GMC – BAU/mL	514.58	3448.32	533.45	3680.14	453.81	2728.26
(95% CI)	(447.84–591.26)	(2949.07–4032.10)	(456.07–623.96)	(3116.64–4345.52)	(333.47–617.56)	(1789.39–4159.74)
Anti-spike IgG GMFR – fold	45.91	262.26	46.40	234.19	44.25	391.25
(95% CI)	(40.12–52.54)	(194.00–354.54)	(39.78–54.11)	(161.96–338.64)	(33.08–59.20)	(258.98–591.08)
Anti-spike IgG SCR – n (%)	208 (98.6)	66 (97.1)	162 (98.8)	51 (96.2)	46 (97.9)	15 (100)
(95% CI)	(95.9–99.7)	(89.8–99.6)	(95.7–99.9)	(87.0–99.5)	(88.7–99.9)	(78.2–100.0)
6 months after the 2nd vaccination (D197)						
PNA – n	193	66	147	51	46	15
NT_{50} GMT – Reciprocal dilution	63.56	142.36	64.77	174.54	59.86	71.20
(95% CI)	(49.78–81.16)	(110.62–183.20)	(49.39–84.92)	(130.44–233.54)	(33.90–105.73)	(50.31–100.75)
NT_{50} GMFR – fold	5.87	12.16	6.07	14.23	5.25	7.12

		Overall		18 – 59 yrs		≥ 60 yrs	
		HXP-GPOVac (N = 224)	BNT162b2 (N = 76)	HXP-GPOVac (N = 174)	BNT162b2 (N = 59)	HXP-GPOVac (N = 50)	BNT162b2 (N = 17)
	(95% CI)	(4.60–7.47)	(9.46–15.63)	(4.64–7.94)	(10.54–19.21)	(2.99–9.22)	(5.03–10.07)
	NT_{50} SCR – n (%)	106 (54.9)	57 (86.4)	82 (55.8)	45 (88.2)	24 (52.2)	12 (80.0)
	(95% CI)	(47.6–62.1)	(75.7–93.6)	(47.4–64.0)	(76.1–95.6)	(36.9–67.1)	(51.9–95.7)
ELISA – n		193	65	147	50	46	15
	Anti-spike IgG GMC – BAU/mL	61.01	237.57	61.37	273.08	59.88	147.97
	(95% CI)	(48.96–76.02)	(194.51–290.17)	(48.09–78.31)	(215.05–346.75)	(35.85–100.00)	(116.54–187.87)
	Anti-spike IgG GMFR – fold	5.52	18.80	5.45	18.13	5.78	21.22
	(95% CI)	(4.51–6.77)	(14.46–24.45)	(4.37–6.79)	(13.00–25.30)	(3.52–9.49)	(15.67–28.74)
	Anti-spike IgG SCR – n (%)	103 (53.4)	61 (93.8)	79 (53.7)	46 (92.0)	24 (52.2)	15 (100.0)
	(95% CI)	(46.1–60.6)	(85.0–98.3)	(45.3–62.0)	(80.8–97.8)	(36.9–67.1)	(78.2–100.0)

Abbreviations: PNA, pseudovirus neutralization assay; NT50, 50% neutralization titer; ELISA, enzyme-linked immunosorbent assay; GMT, geometric mean titer; GMC, geometric mean concentration; GMFR, geometric mean fold rise; SCR, seroconversion rate; BAU/mL, binding antibody units per milliliter; CI, confidence interval; LLOQ, lower limit of quantification; PP, per-protocol; D#, study day (e.g., D1 = Day 1, D29 = Day 29, etc.).
Note: GMTs, GMCs, and GMFRs are calculated from log-transformed values and back-transformed with two-sided 95% CIs. Titers < LLOQ (10) and concentrations < LLOQ (6.3 BAU/mL) were imputed as LLOQ/2. SCR is defined as ≥4-fold rise from baseline; 95% CIs are exact (Clopper–Pearson).

Table S4 – Cell-mediated immune responses to SARS-CoV-2 spike: PBMCs, IFN-γ and IL-5 ELISpot geometric mean concentrations and ratios in HXP-GPOVac and BNT162b2 recipients at baseline (D1), 14 days after the second dose (D43), and 6 months after the second dose (D197), using SARS-CoV-2 peptide vial 1 and vial 2

	Vaccine group	
	HXP-GPOVac	BNT162b2
Baseline (D1)		
PBMCs – n	27	9
GMC (95% CI) – 10^6 cells/mL	6.30 (5.63–7.05)	5.75 (3.74–8.84)
Median (Min, Max) – 10^6 cells/mL	6.00 (4.75, 20.20)	6.01 (1.65, 14.80)
SARS-CoV-2 vial 1		
IFN-gamma ELISpot – n	27	9
GMC (95% CI) – SFU/10^6 cells	11.89 (6.16–22.96)	5.13 (1.81–14.50)
Median (Min, Max) – SFU/10^6 cells	13.33 (0.00, 155.00)	6.67 (0.00, 28.33)
IL-5 ELISpot – n	27	9
GMC (95% CI) – SFU/10^6 cells	23.11 (17.14–31.15)	16.11 (6.73–38.56)
Median (Min, Max) – SFU/10^6 cells	26.67 (0.00, 61.67)	23.33 (0.00, 38.33)
IFN-gamma ELISpot / IL-5 ELISpot ratio – n	27	9
GMC (95% CI) – SFU/10^6 cells	0.51 (0.28–0.95)	0.32 (0.10–1.04)

	Vaccine group	
	HXP-GPOVac	BNT162b2
Median (Min, Max) – SFU/10^6 cells	0.50 (0.03, 7.27)	0.50 (0.03, 2.13)
SARS-CoV-2 vial 2		
IFN-gamma ELISpot – n	27	9
GMC (95% CI) – SFU/10^6 cells	12.30 (6.26–24.17)	5.89 (2.03–17.08)
Median (Min, Max) – SFU/10^6 cells	11.67 (0.00, 353.33)	10.00 (0.00, 28.33)
IL-5 ELISpot – n	27	9
GMC (95% CI) – SFU/10^6 cells	22.04 (16.29–29.80)	19.13 (7.42–49.34)
Median (Min, Max) – SFU/10^6 cells	26.67 (0.00, 48.33)	25.00 (0.00, 66.67)
IFN-gamma ELISpot / IL-5 ELISpot ratio – n	27	9
GMC (95% CI) – SFU/10^6 cells	0.56 (0.29–1.07)	0.31 (0.09–1.08)
Median (Min, Max) – SFU/10^6 cells	0.72 (0.03, 11.16)	0.40 (0.03, 1.89)
14 days after the 2nd vaccination (D43)		
PBMCs – n	27	6
GMC (95% CI) – 10^6 cells/mL	5.84 (5.57–6.13)	5.80 (5.23–6.43)
Median (Min, Max) – 10^6 cells/mL	6.00 (4.75, 20.20)	5.72 (5.26, 6.70)
SARS-CoV-2 vial 1		
IFN-gamma ELISpot – n	27	6
GMC (95% CI) – SFU/10^6 cells	26.59 (11.40–61.99)	17.13 (1.63–179.51)
Median (Min, Max) – SFU/10^6 cells	20.00 (0.00, 1155.00)	50.27 (0.00, 150.00)
IL-5 ELISpot – n	27	6
GMC (95% CI) – SFU/10^6 cells	24.78 (20.34–30.20)	21.75 (17.35–27.28)
Median (Min, Max) – SFU/10^6 cells	26.67 (6.67, 51.67)	20.00 (18.33, 33.33)
IFN-gamma ELISpot / IL-5 ELISpot ratio – n	27	6
GMC (95% CI) – SFU/10^6 cells	1.07 (0.43–2.68)	0.79 (0.06–9.55)
Median (Min, Max) – SFU/10^6 cells	1.00 (0.02, 59.97)	2.41 (0.03, 7.50)
SARS-CoV-2 vial 2		
IFN-gamma ELISpot – n	27	6
GMC (95% CI) – SFU/10^6 cells	28.86 (12.03–69.22)	16.74 (1.51–186.21)
Median (Min, Max) – SFU/10^6 cells	26.67 (0.00, 1308.33)	39.27 (0.00, 231.67)
IL-5 ELISpot – n	27	6
GMC (95% CI) – SFU/10^6 cells	23.01 (18.02–29.38)	22.72 (14.62–35.32)
Median (Min, Max) – SFU/10^6 cells	30.00 (5.00, 58.33)	24.15 (13.33, 41.67)
IFN-gamma ELISpot / IL-5 ELISpot ratio – n	27	6
GMC (95% CI) – SFU/10^6 cells	1.25 (0.51–3.07)	0.74 (0.05–11.44)
Median (Min, Max) – SFU/10^6 cells	1.00 (0.03, 76.33)	2.03 (0.02, 9.93)
6 months after the 2nd vaccination (D197)		
PBMCs – n	26	7
GMC (95% CI) – 10^6 cells/mL	6.46 (6.09–6.86)	5.74 (5.38–6.12)

	Vaccine group	
	HXP-GPOVac	BNT162b2
Median (Min, Max) – 10^6 cells/mL	6.30 (5.16, 8.28)	5.76 (5.00, 6.24)
SARS-CoV-2 vial 1		
IFN-gamma ELISpot – n	26	7
GMC (95% CI) – SFU/10^6 cells	12.36 (6.33–24.14)	9.89 (2.19–44.59)
Median (Min, Max) – SFU/10^6 cells	9.13 (0.00, 320.00)	13.33 (0.00, 185.00)
IL-5 ELISpot – n	26	7
GMC (95% CI) – SFU/10^6 cells	30.14 (24.69–36.79)	29.89 (21.86–40.89)
Median (Min, Max) – SFU/10^6 cells	30.00 (11.67, 68.33)	28.33 (18.33, 48.33)
IFN-gamma ELISpot / IL-5 ELISpot ratio – n	26	7
GMC (95% CI) – SFU/10^6 cells	0.41 (0.22–0.77)	0.33 (0.07–1.65)
Median (Min, Max) – SFU/10^6 cells	0.56 (0.02, 5.65)	0.31 (0.04, 7.40)
SARS-CoV-2 vial 2		
IFN-gamma ELISpot – n	26	7
GMC (95% CI) – SFU/10^6 cells	15.10 (7.52–30.32)	15.73 (3.90–63.44)
Median (Min, Max) – SFU/10^6 cells	14.91 (0.00, 388.33)	8.33 (1.67, 163.33)
IL-5 ELISpot – n	26	7
GMC (95% CI) – SFU/10^6 cells	25.54 (20.89–31.22)	33.12 (21.70–50.54)
Median (Min, Max) – SFU/10^6 cells	28.14 (10.00, 56.67)	33.33 (18.33, 75.00)
IFN-gamma ELISpot / IL-5 ELISpot ratio – n	26	7
GMC (95% CI) – SFU/10^6 cells	0.59 (0.29–1.21)	0.47 (0.11–2.13)
Median (Min, Max) – SFU/10^6 cells	0.79 (0.03, 11.65)	0.36 (0.05, 7.54)

Abbreviations: ELISpot, enzyme-linked immunospot; PBMC, peripheral blood mononuclear cell; IFN-γ, interferon gamma; IL-5, interleukin-5; SFU, spot-forming units; GMC, geometric mean concentration; CI, confidence interval. Note: Responses are summarized as GMCs (95% CI) and medians (min, max). IFN-γ and IL-5 are expressed as SFU/10^6 PBMCs. IFN-γ/IL-5 ratios are summarized as GMCs.

Table S5 – Clinical laboratory hematology results by treatment group (safety population).

Visit	Group	n	Normal n (%)	Grade 1 (Mild) n (%)	Grade 2 (Moderate) n (%)	Grade 3–4 n (%)
**Hemoglobin (g/dL)**						
Baseline	All (N=300)	298	168 (56.4)	76 (25.5)	50 (16.8)	4 (1.3)
	HXP-GPOVac	223	125 (56.1)	57 (25.6)	38 (17.0)	3 (1.3)
	BNT162b2	75	43 (57.3)	19 (25.3)	12 (16.0)	1 (1.3)
Day 8	All (N=300)	300	155 (51.7)	78 (26.0)	58 (19.3)	9 (3.0)
	HXP-GPOVac	224	115 (51.3)	56 (25.0)	45 (20.1)	8 (3.6)
	BNT162b2	76	40 (52.6)	22 (28.9)	13 (17.1)	1 (1.3)
Day 36	All (N=300)	280	105 (37.5)	81 (28.9)	78 (27.9)	16 (5.7)

Visit	Group	n	Normal n (%)	Grade 1 (Mild) n (%)	Grade 2 (Moderate) n (%)	Grade 3–4 n (%)
	HXP-GPOVac	211	75 (35.5)	61 (28.9)	60 (28.4)	15 (7.1)
	BNT162b2	69	30 (43.5)	20 (29.0)	18 (26.1)	1 (1.4)
**White blood cell count (×10³/µL)**						
Baseline	All (N=300)	298	272 (91.3)	26 (8.7)	0 (0.0)	0 (0.0)
	HXP-GPOVac	223	205 (91.9)	18 (8.1)	0 (0.0)	0 (0.0)
	BNT162b2	75	67 (89.3)	8 (10.7)	0 (0.0)	0 (0.0)
Day 8	All (N=300)	300	272 (90.7)	28 (9.3)	0 (0.0)	0 (0.0)
	HXP-GPOVac	224	203 (90.6)	21 (9.4)	0 (0.0)	0 (0.0)
	BNT162b2	76	69 (90.8)	7 (9.2)	0 (0.0)	0 (0.0)
Day 36	All (N=300)	280	252 (90.0)	28 (10.0)	0 (0.0)	0 (0.0)
	HXP-GPOVac	211	190 (90.0)	21 (10.0)	0 (0.0)	0 (0.0)
	BNT162b2	69	62 (89.9)	7 (10.1)	0 (0.0)	0 (0.0)
**Platelets (×10³/µL)**						
Baseline	All (N=300)	298	294 (98.7)	3 (1.0)	1 (0.3)	0 (0.0)
	HXP-GPOVac	223	219 (98.2)	3 (1.3)	1 (0.4)	0 (0.0)
	BNT162b2	75	75 (100.0)	0 (0.0)	0 (0.0)	0 (0.0)
Day 8	All (N=300)	300	299 (99.7)	1 (0.3)	0 (0.0)	0 (0.0)
	HXP-GPOVac	224	223 (99.6)	1 (0.4)	0 (0.0)	0 (0.0)
	BNT162b2	76	76 (100.0)	0 (0.0)	0 (0.0)	0 (0.0)
Day 36	All (N=300)	280	278 (99.3)	1 (0.4)	1 (0.4)	0 (0.0)
	HXP-GPOVac	211	210 (99.5)	1 (0.5)	0 (0.0)	0 (0.0)
	BNT162b2	69	68 (98.6)	0 (0.0)	1 (1.4)	0 (0.0)

Abbreviations: DAIDS, Division of AIDS; n, number of participants with evaluable result at the specified visit.

Note: Data are n (%) of evaluable participants. Severity grading per the Division of AIDS (DAIDS) Table for Grading the Severity of Adult and Pediatric Adverse Events, Version 2.1 (2017). Hemoglobin Grade 1 (Mild): Male 12.5–13.5 g/dL, Female 11.0–12.0 g/dL; Grade 2 (Moderate): Male 10.5–12.4 g/dL, Female 9.5–10.9 g/dL; Grade 3 (Severe): Male 8.5–10.4 g/dL, Female 8.0–9.4 g/dL; Grade 4: Male <8.5 g/dL, Female <8.0 g/dL. WBC Grade 1: decreased 2,500–3,400 or increased 10,800–15,000 cells/mm³; Grades 2–4: progressively lower or higher counts. Platelets Grade 1: decreased 125,000–140,000 cells/mm³. Two participants did not have baseline hematology results available. No hematology abnormality was assessed as clinically significant or related to vaccination. The denominator for Day 36 is 280 (HXP-GPOVac: 211; BNT162b2: 69).

Table S6 – Clinical laboratory serum chemistry results by treatment group (safety population).

Visit	Group	n	Normal n (%)	Grade 1 (Mild) n (%)	Grade 2 (Moderate) n (%)	Grade 3–4 n (%)
**Creatinine (mg/dL)**						
Baseline	All (N=300)	300	297 (99.0)	3 (1.0)	0 (0.0)	0 (0.0)
	HXP-GPOVac	224	221 (98.7)	3 (1.3)	0 (0.0)	0 (0.0)
	BNT162b2	76	76 (100.0)	0 (0.0)	0 (0.0)	0 (0.0)
Day 8	All (N=300)	300	293 (97.7)	5 (1.7)	2 (0.7)	0 (0.0)
	HXP-GPOVac	224	218 (97.3)	4 (1.8)	2 (0.9)	0 (0.0)
	BNT162b2	76	75 (98.7)	1 (1.3)	0 (0.0)	0 (0.0)
Day 36	All (N=300)	280	276 (98.6)	4 (1.4)	0 (0.0)	0 (0.0)
	HXP-GPOVac	211	208 (98.6)	3 (1.4)	0 (0.0)	0 (0.0)
	BNT162b2	69	68 (98.6)	1 (1.4)	0 (0.0)	0 (0.0)
**Alanine aminotransferase / ALT (U/L)**						
Baseline	All (N=300)	300	278 (92.7)	22 (7.3)	0 (0.0)	0 (0.0)
	HXP-GPOVac	224	211 (94.2)	13 (5.8)	0 (0.0)	0 (0.0)
	BNT162b2	76	67 (88.2)	9 (11.8)	0 (0.0)	0 (0.0)
Day 8	All (N=300)	300	274 (91.3)	24 (8.0)	2 (0.7)	0 (0.0)
	HXP-GPOVac	224	208 (92.9)	15 (6.7)	1 (0.4)	0 (0.0)
	BNT162b2	76	66 (86.8)	9 (11.8)	1 (1.3)	0 (0.0)
Day 36	All (N=300)	280	250 (89.3)	26 (9.3)	4 (1.4)	0 (0.0)
	HXP-GPOVac	211	191 (90.5)	17 (8.1)	3 (1.4)	0 (0.0)
	BNT162b2	69	59 (85.5)	9 (13.0)	1 (1.4)	0 (0.0)
**Aspartate aminotransferase / AST (U/L)**						
Baseline	All (N=300)	300	273 (91.0)	26 (8.7)	1 (0.3)	0 (0.0)
	HXP-GPOVac	224	203 (90.6)	20 (8.9)	1 (0.4)	0 (0.0)
	BNT162b2	76	70 (92.1)	6 (7.9)	0 (0.0)	0 (0.0)
Day 8	All (N=300)	300	260 (86.7)	39 (13.0)	1 (0.3)	0 (0.0)
	HXP-GPOVac	224	194 (86.6)	30 (13.4)	0 (0.0)	0 (0.0)
	BNT162b2	76	66 (86.8)	9 (11.8)	1 (1.3)	0 (0.0)
Day 36	All (N=300)	280	239 (85.4)	35 (12.5)	5 (1.8)	1 (0.4)
	HXP-GPOVac	211	180 (85.3)	27 (12.8)	4 (1.9)	0 (0.0)
	BNT162b2	69	59 (85.5)	8 (11.6)	1 (1.4)	1 (1.4)
**Total bilirubin (mg/dL)**						
Baseline	All (N=300)	300	293 (97.7)	7 (2.3)	0 (0.0)	0 (0.0)

Visit	Group	n	Normal n (%)	Grade 1 (Mild) n (%)	Grade 2 (Moderate) n (%)	Grade 3–4 n (%)
	HXP-GPOVac	224	218 (97.3)	6 (2.7)	0 (0.0)	0 (0.0)
	BNT162b2	76	75 (98.7)	1 (1.3)	0 (0.0)	0 (0.0)
Day 8	All (N=300)	300	293 (97.7)	5 (1.7)	1 (0.3)	1 (0.3)
	HXP-GPOVac	224	220 (98.2)	3 (1.3)	1 (0.4)	0 (0.0)
	BNT162b2	76	73 (96.1)	2 (2.6)	0 (0.0)	1 (1.3)
Day 36	All (N=300)	280	270 (96.4)	7 (2.5)	3 (1.1)	0 (0.0)
	HXP-GPOVac	211	205 (97.2)	5 (2.4)	1 (0.5)	0 (0.0)
	BNT162b2	69	65 (94.2)	2 (2.9)	2 (2.9)	0 (0.0)

Abbreviations: ALT, alanine aminotransferase; AST, aspartate aminotransferase; DAIDS, Division of AIDS; n, number of participants with evaluable result at the specified visit; ULN, upper limit of normal.

Note: Data are n (%) of evaluable participants. Severity grading per the Division of AIDS (DAIDS) Table for Grading the Severity of Adult and Pediatric Adverse Events, Version 2.1 (2017). Creatinine: Grade 1, 1.1–1.3 × ULN; Grade 2, >1.3–1.8 × ULN. ALT and AST: Grade 1, 1.25–<2.5 × ULN; Grade 2, 2.5–<5.0 × ULN; Grade 3, 5.0–<10.0 × ULN; Grade 4, ≥10.0 × ULN. Total bilirubin: Grade 1, 1.1–<1.6 × ULN; Grade 2, 1.6–<2.6 × ULN; Grade 3, 2.6–<5.0 × ULN. One participant in the BNT162b2 group had a Grade 3 bilirubin value at Day 8 (assessed as not clinically significant and not related to vaccination). One participant in the BNT162b2 group had a Grade 3 AST value at Day 36 (assessed as not clinically significant and not related to vaccination). The denominator for Day 36 is 280 (HXP-GPOVac: 211; BNT162b2: 69).
